# Water Table and Vegetation Shape Peatland CO_2_ and CH_4_ Fluxes With Water Table Modulating Temperature Sensitivity

**DOI:** 10.1111/gcb.71129

**Published:** 2026-09-27

**Authors:** Heyu Chen, Jirateep Yotmaw, Liam Thompson, Dominik Bittner, Yongxing Ren, Yi Xu, Scott J. Davidson, Jingui Wei, Wen Yin, Grant Campbell, Shuai Xue, Chris D. Evans, Pete Smith, Astley Hastings, Mohamed Abdalla

**Affiliations:** ^1^ Institute of Biological and Environmental Sciences University of Aberdeen Aberdeen UK; ^2^ State Key Laboratory of Black Soils Conservation and Utilization, Northeast Institute of Geography and Agroecology Chinese Academy of Sciences Changchun China; ^3^ Jiangsu Provincial University Key Laboratory of Agricultural and Ecological Meteorology, School of Ecology and Applied Meteorology Nanjing University of Information Science & Technology Nanjing China; ^4^ Département Des Sciences Biologiques Université du Québec à Montréal Montréal Quebec Canada; ^5^ State Key Laboratory of Aridland Crop Science, College of Agronomy Gansu Agricultural University Lanzhou China; ^6^ Hunan Engineering Laboratory of Miscanthus Ecological Applications, College of Bioscience & Biotechnology Hunan Agricultural University Changsha China; ^7^ UK Centre for Ecology & Hydrology Environment Centre Wales Bangor UK

**Keywords:** climate feedback, earth system models, plant functional type, temperature sensitivity (Q_10_), water‐table level, wetland carbon balance

## Abstract

Peatland rewetting can reduce CO_2_ loss but increase CH_4_ emissions. How vegetation shapes this trade‐off and how the water table modifies temperature sensitivity remain unclear. We analysed 14,643 observations from 285 peatland sites. CH_4_ emissions increased more steeply with rising water‐table level (WTL) under graminoid dominance, while the pattern of declining ecosystem respiration differed between vegetation classes. Combined CO_2_‐equivalent emissions (GHGE; respiration + CH_4_ at GWP_100_ = 28) declined by 73.9% as WTL rose from 60 cm below to 15 cm above the peat surface and were still declining at the wet end of the analysed range. The decline persisted under GWP_20_, although the magnitude of the decline above the peat surface depended strongly on the CH_4_ weighting. The within‐site analysis further showed that apparent CH_4_ temperature sensitivity (Q_10_) increased by 14.9% per 10 cm rise in WTL (95% CI 6.9%–23.5%), whereas respiration Q_10_ showed no detectable WTL dependence. A supplementary comparison within non‐graminoid‐dominated bogs showed that fitted CH_4_ Q_10_ increased with WTL where graminoids were present and decreased where graminoids were absent or negligible. Under +3°C warming, fixing CH_4_ Q_10_ at its value at WTL = −10 cm gave a predicted warming multiplier that was 24.7% larger at WTL = −63 cm and 11.8% smaller at +20 cm than the WTL‐dependent formulation. These results suggest that assessments of peatland rewetting under warming should consider both vegetation differences in emission responses and the WTL dependence of CH_4_ temperature sensitivity.

## Graphical abstract

Using 14,643 chamber observations from 285 peatland sites, we found that wetter conditions were associated with higher CH_4_ emissions but lower ecosystem respiration and combined CO_2_‐equivalent emissions. Apparent CH_4_ temperature sensitivity also increased with water‐table level, suggesting that fixed Q_10_ values may misrepresent warming responses.

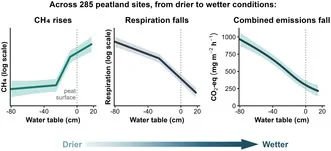

## 1 Introduction

Peatlands cover only ~3% of the global land surface but store nearly one‐third of global soil carbon in waterlogged deposits accumulated over millennia (UNEP 2022; Yu et al. 2010). These carbon stocks are often considered irrecoverable because they cannot be re‐sequestered within timescales relevant to near‐term climate targets after release (Goldstein et al. 2020). Their long‐term preservation depends on sustained waterlogged conditions. Anoxia suppresses aerobic decomposition and ecosystem respiration (*R*_eco_) but also promotes methane (CH_4_) production (Frolking et al. 2006). Wetlands are the largest natural source of atmospheric CH_4_ (Bridgham et al. 2013; Saunois et al. 2020). Given that these emissions pre‐date humanity, they cannot be considered to contribute to anthropogenic climate change (Evans and Gauci 2023), but land‐use or climate change feedbacks leading to increased surface inundation, or biogeochemical modifications such as nutrient enrichment, may result in above‐natural wetland CH_4_ emissions. Drainage also alters the natural carbon cycle by exposing protected subsurface peat carbon to aerobic decomposition at rates that can greatly exceed long‐term peat accumulation rates. Annual greenhouse‐gas (GHG) emissions from drained peatlands have been estimated at approximately 1.91 Gt CO_2_‐eq (Leifeld and Menichetti 2018), equivalent to about 5% of global anthropogenic emissions (Friedlingstein et al. 2019). Rewetting can limit these CO_2_ losses, but the accompanying increase in CH_4_ emissions also needs to be considered.

This CO_2_–CH_4_ trade‐off depends strongly on water‐table level (WTL). Raising WTL restricts aerobic decomposition but can promote CH_4_ production and decrease CH_4_ oxidation before it reaches the atmosphere (Evans et al. 2021; Zhang et al. 2024). Previous studies have therefore sought WTL ranges that minimise net GHG emissions. Several studies have proposed low‐emission ranges near or below the peat surface (Beetz et al. 2013; Evans et al. 2021; Koch et al. 2023; Zhao et al. 2026). These findings provide a basis for restoration practice. However, their applicability across different vegetation classes and thermal conditions remains uncertain. Current IPCC Tier 1 emission factors for organic soils also provide limited representation of variation in WTL and vegetation composition within broad land‐use and management categories (Wilson et al. 2016).

Evans et al. (2021) provided a key quantitative basis for peatland rewetting by identifying a low‐emission water‐table range from a synthesis of UK and Irish flux measurements supplemented by published eddy‐covariance data from other temperate and boreal peatlands. They quantified the overall peatland CO_2_ balance using net ecosystem production, which includes gross primary productivity (GPP) and *R*_eco_. Their analysis also included harvested carbon exports where relevant. Our analysis focuses on an emission‐side GHG equivalent (GHGE) combining *R*_eco_ and CH_4_. This metric allows us to examine how combined *R*_eco_ and CH_4_ emissions respond to WTL.

Vegetation composition and soil temperature can modify flux–WTL relationships (Bao et al. 2021; Bridgham et al. 2013). Vegetation influences gas transport and substrate supply, while soil temperature affects reaction rates. Vegetation can also affect *R*_eco_ through plant respiration and the supply of recent photosynthate to soil microorganisms (Ward et al. 2009). Many graminoid species possess aerenchyma that transports CH_4_ directly from soil to atmosphere, bypassing some of the aerobic oxidation zone (Ström et al. 2012; Whiting and Chanton 1992). Graminoid root exudates can also deliver labile carbon to anaerobic soil and provide substrate for methanogens (Strack et al. 2004; Waldo et al. 2019). Under drought, some graminoid species also release ethylene that can inhibit CH_4_ oxidation by methanotrophs (Zhou et al. 2021). These traits may help explain why CH_4_ responds differently to WTL across vegetation communities.

These vegetation effects may also influence temperature sensitivity (Q_10_). Apparent Q_10_ reflects both intrinsic reaction kinetics and constraints on substrate supply (Davidson et al. 2006; Yvon‐Durocher et al. 2012). A peat mesocosm study found that the presence of *Molinia caerulea* decreased the temperature sensitivity of *R*_eco_ but increased that of CH_4_ emissions (Leroy et al. 2017). Changes in WTL alter oxygen conditions and substrate availability and may therefore modify the apparent temperature response of CH_4_ emissions (Bridgham et al. 2013; Davidson et al. 2006). Plant transport and substrate inputs may further modify this response. We therefore examined how apparent Q_10_ varied with WTL for both CH_4_ and *R*_eco_. We also tested whether this hydrological dependence differed between graminoid‐dominated (GRA) and non‐graminoid‐dominated (NGRA) peatlands.

Land‐surface models represent temperature effects through response functions for processes such as respiration and methanogenesis. CH_4_ models such as CLM4Me and ORCHIDEE‐PEAT simulate production, oxidation and transport, with net emissions emerging from these interacting processes (Riley et al. 2011; Salmon et al. 2022). A fixed temperature parameter for an individual process does not necessarily imply a fixed apparent Q_10_ for net CH_4_ emissions. Models of plant and soil respiration also allow temperature sensitivity to vary with temperature or soil moisture (Kim et al. 2019; Tjoelker et al. 2001). At the ecosystem scale, Niu et al. (2021) projected that the apparent Q_10_ of ecosystem respiration would decrease under warming. However, how WTL modifies apparent Q_10_ for both CH_4_ and *R*_eco_ remains poorly resolved in peatlands. Quantifying this relationship could help assess fixed‐Q_10_ assumptions and provide an observational benchmark for models. Bao et al. (2021) also suggested that vegetation and water level may jointly influence wetland Q_10_. Testing these effects requires observations that resolve both hydrological conditions and vegetation differences. Chamber measurements link fluxes to local water levels and vegetation communities and are therefore useful for examining these relationships.

Here, we compiled a global static‐chamber database of 14,643 observations from 285 sites within 100 peatland complexes. We asked three questions. First, how do CH_4_, *R*_eco_, and their combined emission‐side GHGE vary with WTL and dominant vegetation? Second, does apparent Q_10_ vary systematically with WTL, and does this dependence differ between vegetation classes? Third, how do warming responses estimated using a fixed Q_10_ compare with those estimated using a WTL‐dependent Q_10_?

## 2 Data and Methods

### 2.1 Database Compilation

#### 2.1.1 Search Terms

We compiled the wetland carbon‐flux database from published studies and open‐access data repositories. Peer‐reviewed studies were identified through Web of Science (August 2025) using combined search terms across three domains: ecosystem type (wetland OR peatland OR swamp OR marsh OR fen OR bog OR wet tundra OR wet meadow), hydrological driver (water table OR water level OR water depth), and carbon‐flux metric (GHG OR soil carbon flux OR CO_2_ OR ecosystem respiration OR soil respiration OR CH_4_ OR methane). We further integrated data from the published global wetland CO_2_–CH_4_ flux database compiled by Chen et al. (2021), the Arctic–Boreal Carbon Flux database (ABCFlux v2), the Arctic Data Center (with emphasis on Alaskan peatland records), PANGAEA and the UKCEH Environmental Information Data Centre. To prevent pseudo‐replication between published studies and repository datasets, records were deduplicated by cross‐checking site coordinates, sampling dates, and principal investigator metadata.

#### 2.1.2 Inclusion Criteria

The compiled dataset was filtered using four criteria. First, only in situ field observations were retained; laboratory incubations and mesocosm experiments were excluded. Second, primary CO_2_ flux records were restricted to opaque (dark) chamber measurements, excluding photosynthetic CO_2_ uptake during measurement. These CO_2_ fluxes therefore represent *R*_eco_. A parallel subset of paired light–dark observations with simultaneous GPP estimates was retained separately for the GPP–WTL sensitivity analysis described below. Third, records used in the flux–WTL analyses were required to contain gas flux (CH_4_ and/or CO_2_) and WTL at matched temporal resolution. Fourth, observations from nitrogen addition or biochar amendment treatments were excluded to limit the influence of these experimental amendments on the measured fluxes. To restrict the analysis to peatlands, we applied a two‐step site classification. First, sites explicitly described as peatlands in the source, or for which the source reported a peat layer, were assigned to the peatland subset directly. For sites without such an explicit description, we used the reported soil classification, and sites with soil types corresponding to common peat soils, such as Histosols, were classified as peatlands.

#### 2.1.3 Vegetation Classification

Conventional ecosystem labels (e.g., bog vs. fen) can combine sites with different vegetation structure and functional traits. We therefore classified vegetation according to graminoid dominance. Graminoids were used as a functional descriptor because many sedges, grasses and reeds possess aerenchymatous tissues that can contribute to plant‐mediated CH_4_ transport and rhizosphere processes (Couwenberg et al. 2011; Ward et al. 2013). Records labelled only as graminoid‐present or lacking a vegetation label, and other records from the same peatland complexes, were re‐checked against the original publications (107 of 316 vegetation records; Table S9). Where the available information allowed graminoid presence to be resolved, observations were assigned to one of three states: graminoid dominant, graminoid present but not dominant, and graminoid absent or negligible. The primary vegetation comparison was based on dominance. Graminoid‐dominated observations were assigned to GRA, whereas observations dominated by mosses, shrubs, trees or other non‐graminoid vegetation were assigned to NGRA, regardless of whether graminoids were also present. Graminoid presence was retained as a separate descriptor and was used only in secondary analyses testing whether the presence of graminoids provided additional information beyond dominance. Observations with known vegetation dominance but unresolved graminoid presence were retained in the dominance‐based analyses and excluded from the presence‐based comparisons. Observations whose vegetation dominance could not be resolved, or whose vegetation was recorded as mixed with no dominant group, were excluded from all vegetation comparisons. Within the analysis domain, these included 734 observations from 21 sites. Vegetation class was assigned at the measurement‐site level and applied to all observations from that site, except where the source described different vegetation for separate microforms, plots or sampling locations, which were classified separately. The vegetation class assigned to every site is listed in Table S9, and its source, confidence and any reassignment are released with the dataset.

#### 2.1.4 Statistical Weighting and the Treatment of Extreme Fluxes

Data sources differed in their level of spatial replication. Literature records often represented means of several replicates, whereas repository records often contained individual observations. We assigned each observation a weight of *W*_*i*_ = ln(*N*_*i*_) + 1, where *N*_*i*_ is the number of spatial replicates. This weighting gives greater influence to means based on more spatial replicates while preventing highly replicated observations from dominating the analysis. Unweighted fits and square‐root weighting were examined as sensitivity analyses (Figure S9). For the flux–WTL models, fluxes (mg CH_4_ m^−2^ h^−1^ and mg CO_2_ m^−2^ h^−1^) were transformed as slog(*x*) = sign(*x*) × ln (1 + |*x*|). This transformation reduces skewness while retaining zero and negative fluxes. All eligible observations were retained in the primary analyses. Removal of extreme values using 1.5× and 3× interquartile range (IQR) rules, together with winsorisation, was evaluated separately (Table S7). These treatments were used only as sensitivity analyses because removal rates varied along the WTL gradient.

#### 2.1.5 Database Composition and Analysis Domains

The final peatland database contained 14,643 observations from 285 measurement sites within 100 peatland complexes, of which 13,599 observations from 278 sites contained WTL together with CH_4_ or *R*_eco_. A peatland complex is a named peatland, locality or research area containing one or more measurement sites. The measurement site remains the sampling unit throughout, and the complex identifies sites that belong to the same broader geographical location. WTL was expressed in centimetres relative to the peat surface, with negative values below the surface and positive values above it. Within the flux–WTL analysis domain of −80 to +30 cm, 12,305 CH_4_ observations from 242 sites and 5530 *R*_eco_ observations from 158 sites were available. The emission‐side GHGE analysis used 4556 paired CH_4_–*R*_eco_ observations from 120 sites. A paired CH_4_–*R*_eco_ observation was defined as a database record that reported both CH_4_ and *R*_eco_ fluxes for the same site and sampling period, together with the corresponding WTL. The spatial distribution and vegetation‐classification framework are shown in Figure 1. The database and the R script that reproduces the main results are archived on Zenodo (Chen et al. 2026).

**FIGURE 1:**
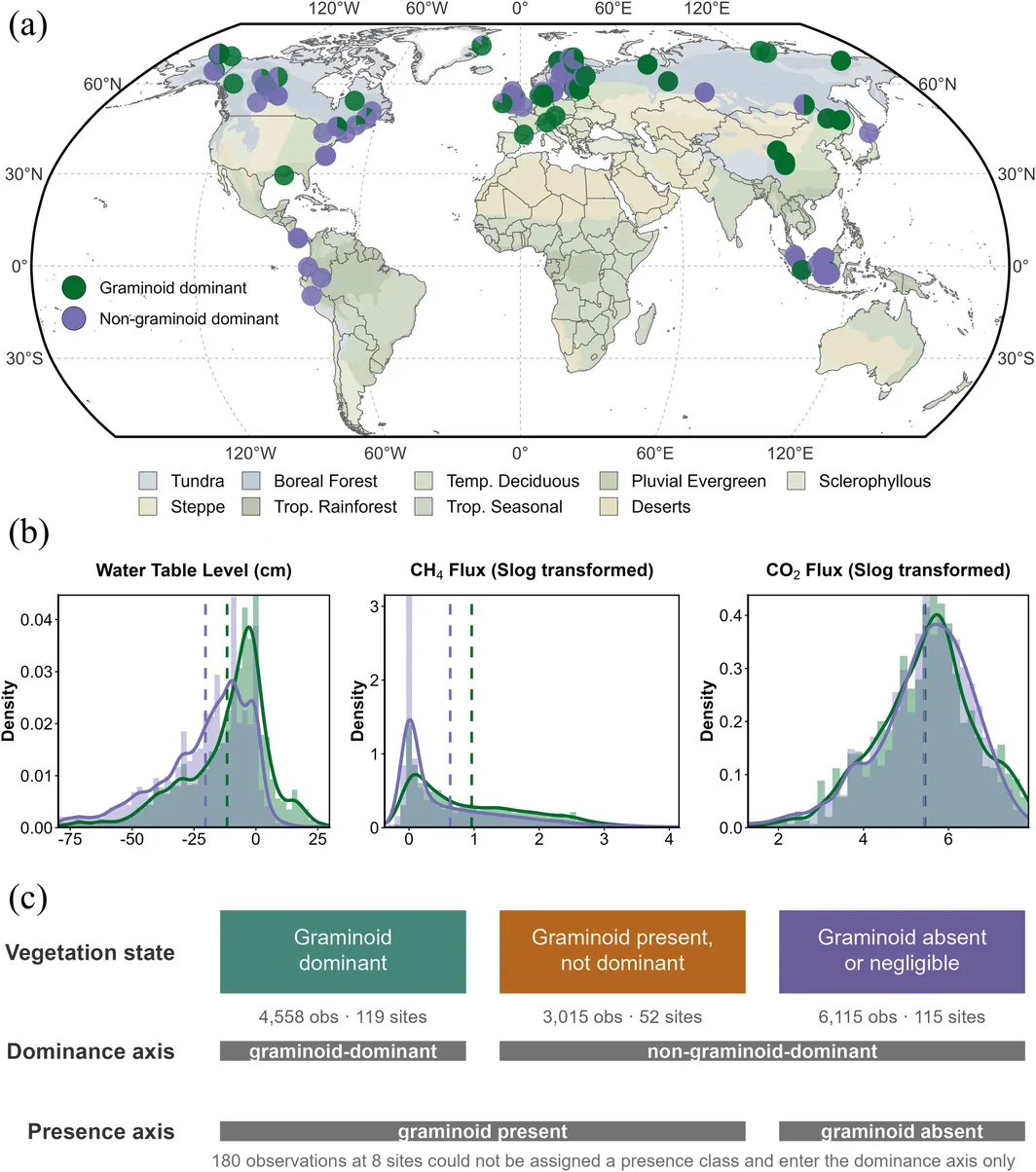
Spatial distribution and statistical characteristics of the peatland dataset. (a) Global distribution of 285 flux measurement sites across 100 peatland complexes, overlaid on global biome types. Each symbol marks one location (178 locations; co‐located sites share a symbol), and its segments show the proportion of observations in each dominance class. A total of 119 site names include graminoid‐dominated observations and 167 include non‐graminoid‐dominated observations. Eighteen site names contribute observations to both classes because they include different microforms, plots or sampling locations, and the remaining 17 of the 285 sites have no resolved dominance class and are not shown on the map. (b) Density distributions of water‐table level (WTL; *n* = 13,301 observations within the −80 to +30 cm analysis domain), signed‐log‐transformed CH_4_ flux (*n* = 11,717 observations) and signed‐log‐transformed ecosystem respiration (*R*_eco_) flux (*n* = 5252), shown by dominant vegetation class. Observations without a resolved dominance class are not shown. Fluxes were expressed in mg CH_4_ m^−2^ h^−1^ and mg CO_2_ m^−2^ h^−1^ before transformation. Histograms display the observed distributions, solid curves are smoothed density estimates and vertical dashed lines indicate group means. (c) Vegetation classification framework derived from the source descriptions. Vegetation information was organised into three states: Graminoid dominant, graminoid present but not dominant, and graminoid absent or negligible. The dominance axis contrasts graminoid‐dominated and non‐graminoid‐dominated observations, whereas the presence axis contrasts observations with graminoids present against those with graminoids absent or negligible. Observation and site counts appear beneath each state. The three states include 263 unique site names, with overlapping membership giving a summed count of 286. Another 180 observations from eight sites lack a graminoid‐presence class and contribute only to the dominance axis. These include five additional sites, bringing the total with the 17 unclassified sites in (a) to 285.

#### 2.1.6 Datasets Used for the GPP–WTL Sensitivity Test

The GPP–WTL sensitivity analysis used two datasets. The first contained 1384 paired light–dark chamber observations with simultaneous GPP estimates. The second comprised records from 74 wetland eddy‐covariance sites, of which 61 were retained after quality screening (Table S8). Tower sites were classified using the same dominance‐based vegetation framework as the chamber dataset and assigned a management or ecosystem category: natural, drained, restored, pasture, paddy or tidal. Chamber and eddy‐covariance data were analysed separately because they represent different spatial footprints. The class‐specific eddy‐covariance GPP models also supplied the GPP–WTL coefficient for the exploratory correction described in Section 2.2.6.

### 2.2 Statistical Analysis

All statistical analyses were conducted in R version 4.4.1, except the eddy‐covariance GPP models, which were fitted in Python using statsmodels (version 0.14.6). Linear mixed‐effects models, including the breakpoint models, were fitted with lme4 (version 1.1‐35.5). Breakpoint grid searches and profile‐likelihood support intervals were implemented in custom R code, and models were compared using the Akaike information criterion (AIC) and likelihood‐ratio tests. Marginal and conditional *R* ^2^ values were calculated with MuMIn (version 1.48.4). Generalised additive mixed models, including the hierarchical within‐site Q_10_ models, were fitted with mgcv (version 1.9‐1). Figures were produced with ggplot2 (version 4.0.2).

#### 2.2.1 Nonlinear Hydrological Response Modelling

CH_4_ and *R*_eco_ responses to WTL were fitted using weighted linear mixed‐effects models across the whole dataset and separately for GRA and NGRA peatlands. Models included a site random intercept and used the weights defined in Section 2.1.4. Five response forms were compared by AIC: linear, exponential, quadratic, one‐breakpoint, and two‐breakpoint models. The retained model was chosen within the linear, one‐breakpoint, and two‐breakpoint sequence, and the exponential and quadratic forms were fitted for comparison only. Breakpoint positions were estimated by iterative grid search. Additional breakpoint structure was retained only when it reduced AIC by more than 10 relative to the simpler model (Figure S1). This rule applied only to breakpoint selection. Breakpoint uncertainty was described using profile‐likelihood support intervals. We used likelihood‐ratio tests between nested models to evaluate the vegetation main effect and the vegetation × WTL interaction separately. The interaction test assessed whether the flux–WTL relationship differed between vegetation classes. Model diagnostics and sensitivity analyses are reported in the Supporting Information. Potential small‐study or reporting effects were explored using a funnel plot of site‐level CH_4_ sensitivity slopes against their precision (Figure S11).

#### 2.2.2 Hydrological Modulation of Temperature Sensitivity (Q_10_)

Apparent temperature sensitivity was estimated using hierarchical models that separated within‐site and between‐site temperature variation. Soil temperature was decomposed into a within‐site deviation, $T_{w,\mathrm{ij}}=T_{\mathrm{ij}}-{\bar{T}}_{i}$, and the corresponding site mean, ${\bar{T}}_{i}$, computed using the same ln(*N*) + 1 weights applied in the models. Both temperature components were included in the models. This separation allowed the within‐site temperature response to be estimated while accounting for differences in mean temperature among sites. The within‐site temperature deviation therefore provided the basis for estimating apparent temperature sensitivity, while the site mean temperature accounted for between‐site temperature differences, which can be confounded with differences in climate, vegetation and peatland type. Models included WTL, its interaction with the within‐site temperature deviation, site‐specific random intercepts and random slopes for the within‐site temperature deviation. Apparent Q_10_ at a given WTL was calculated as $Q_{10}=\exp\left(10\beta_{T}\right)$, where $\beta_{T}$ is the fitted within‐site temperature coefficient at WTL. Three prespecified vegetation structures were compared. COMMON used the same Q_10_ level and WTL modulation for GRA and NGRA. REDUCED allowed vegetation‐specific Q_10_ levels with a shared WTL modulation. FULL allowed both Q_10_ levels and WTL modulation to differ between classes. Models were fitted by maximum likelihood and compared using AIC.

Q_10_ models were fitted to the natural logarithm of positive fluxes. Zero and negative observations were excluded from these models but retained in the signed‐log flux–WTL analyses. Negative CH_4_ fluxes were treated as net uptake. Because the frequency of non‐positive CH_4_ observations varied with WTL, we also fitted a signed‐log model using all eligible observations and including the within‐site temperature × WTL interaction. This analysis tested the direction of the interaction and was not interpreted as a Q_10_ estimate. Thus, the CH_4_ Q_10_ estimates describe the temperature sensitivity of positive net CH_4_ emissions rather than the full distribution of net CH_4_ exchange.

Estimates from 30‐cm sliding windows are shown in Figure S10 for descriptive comparison. They were not used for formal inference because neighbouring windows overlap and the contributing sites change along the WTL gradient. We also evaluated a flexible model in which the within‐site temperature coefficient varied smoothly with WTL. Q_10_ curves were presented only where at least 20 sites provided informative within‐site temperature variation. A site was considered informative within a 30‐cm neighbourhood if it contributed at least three observations spanning at least 2°C in soil temperature. This requirement was applied separately to each vegetation class and to the combined vegetation subset, evaluated at 1‐cm steps between −60 and +20 cm and between −80 and +30 cm, respectively. These requirements were set so that Q_10_ curves were presented only over WTL ranges supported by within‐site temperature variation, rather than extrapolated beyond them. Absolute Q_10_ levels were parameterised at WTL = 0 cm. Relative Q_10_ curves were normalised at WTL = −10 cm. The fixed‐Q_10_ comparison is defined in Section 2.2.3.

An exploratory analysis was restricted to NGRA bogs and compared observations with graminoids present against those with graminoids absent or negligible. We used the same within‐site temperature framework to estimate group‐specific WTL modulation of apparent Q_10_. The contrast was expressed as the ratio of the groups' Q_10_ multipliers over a 30 cm rise in WTL. Relative curves were shown over their common WTL support. Sensitivity analyses assessed the contrast using peatland complexes as the clustering unit and by omitting each complex in turn (Table S4).

#### 2.2.3 Q_10_ Parameterisation and Warming‐Response Comparison

For model implementation, hydrological modulation of apparent Q_10_ was expressed as $Q_{10}\left(\mathrm{WTL}\right)=Q_{10}\left(0\right)\times r^{\mathrm{WTL}/10}$, where $Q_{10}\left(0\right)$ is the apparent Q_10_ at the peat surface (WTL = 0 cm) and r is the multiplicative change per 10 cm rise in WTL. For CH_4_, we used the shared modulation from the REDUCED model while retaining vegetation‐specific reference levels. For *R*_eco_, the simplified implementation used *r* = 1 because no WTL dependence was detected. Flexible and linear forms of the within‐site temperature coefficient were compared using AIC. We compared CH_4_ warming flux multipliers calculated using WTL‐dependent Q_10_ with those calculated using Q_10_ fixed at WTL = −10 cm. For a warming increment of ΔT, the flux multiplier was calculated as: $Q_{10}^{\frac{\Delta T}{10}}$. The relative difference was calculated as: $D\left(\mathrm{WTL},\Delta T\right)={\left[\frac{Q_{10}\left(-10\right)}{Q_{10}\left(\mathrm{WTL}\right)}\right]}^{\frac{\Delta T}{10}}-1$. Comparisons used warming increments of +1 and +3°C, and 100× *D* was reported as a percentage. Positive values indicate a larger predicted multiplier under the fixed‐Q_10_ formulation, whereas negative values indicate a smaller multiplier. WTL = −10 cm defines the fixed comparator, so its choice affects the magnitude of the comparison. Uncertainty bands propagated uncertainty in the CH_4_ scaling coefficient conditional on the linear implementation.

#### 2.2.4 Emission‐Side Greenhouse‐Gas Equivalent

GHGE was calculated for each paired observation as *R*_eco_ + *w* × CH_4_. The primary analysis used *w* = 28, the 100‐year global warming potential for biogenic CH_4_, and the alternative IPCC AR5 weights *w* = 34 (100‐year horizon including climate–carbon feedbacks) and *w* = 84 (20‐year horizon) were used to test sensitivity to CH_4_ weighting (Figure S2). GHGE responses were fitted directly to paired observations in CO_2_‐equivalent units (mg CO_2_‐eq m^−2^ h^−1^) using generalised additive mixed models with penalised regression splines, a site random effect and the observation weights defined in Section 2.1.4. Models were fitted across the whole dataset and separately by dominant vegetation class. GHGE curves were not reconstructed from the separate CH_4_ and *R*_eco_ models. The metric excludes photosynthetic uptake and lateral carbon fluxes and therefore describes emissions rather than the full ecosystem GHG balance.

Fitted curves were presented up to the 99th percentile of the corresponding paired WTL distribution. For the whole dataset, GHGE change was evaluated from −60 cm, close to the fifth percentile, to +15 cm, the 99th‐percentile upper limit. Above‐surface changes were evaluated from 0 to +15 cm for the whole dataset and GRA peatlands, and from 0 to +10.4 cm for NGRA peatlands. Percentage change was calculated relative to the fitted GHGE at the starting WTL of each comparison. Curves were examined for an internal minimum within the analysed range. A minimum at the range boundary was not interpreted as an optimal restoration WTL.

#### 2.2.5 Hydrology–Temperature Response Visualisation

To visualise the joint distribution of fluxes across hydrological and thermal conditions, CH_4_ and *R*_eco_ observations were summarised separately for GRA and NGRA peatlands across the WTL × soil‐temperature space. Observations were restricted to WTL = −65 to +25 cm and binned into a 20 × 20 grid. Mean flux within each bin was calculated on the signed‐log scale, and bins without observations were left blank. For visualisation, colour scales were clipped at the 2nd and 98th percentiles of the binned means, with values beyond these limits shown using the corresponding end colours. These heatmaps were used for visualisation. Formal inference on the hydrological modulation of temperature sensitivity was based on the hierarchical within‐site Q_10_ models described in Section 2.2.2.

#### 2.2.6 Parallel GPP–WTL Sensitivity Test

GPP–WTL relationships were tested independently in the chamber and eddy‐covariance datasets described in Section 2.1.6. Chamber GPP was analysed using the hydrological‐response framework applied to *R*_eco_. Eddy‐covariance GPP was modelled as a function of WTL and soil temperature, with a site random intercept, separately within each vegetation class and over WTL = −60 to +20 cm. These models used 48 of the 61 retained towers with concurrent GPP, WTL and soil‐temperature records within this range (33 GRA and 15 NGRA; Table S8).

For the exploratory GPP correction, the WTL coefficients of the two class‐specific eddy‐covariance GPP models were averaged using weights proportional to the number of paired CH_4_–*R*_eco_ observations in each class, to approximate the vegetation composition of the GHGE dataset. The WTL‐dependent component of the fitted GPP response was subtracted from the GHGE model described in Section 2.2.4 as $g_{\mathrm{WTL}}=\beta_{\mathrm{WTL}}\times \mathrm{WTL}$, where $\beta_{\mathrm{WTL}}$ is this weighted mean (−1.52 mg CO_2_ m^−2^ h^−1^ cm^−1^; Figure S8). The correction addressed the WTL‐dependent component of GPP and did not reconstruct the full ecosystem carbon balance.

## 3 Results

### 3.1 Distinct CH_4_ and *R*_eco_ Water‐Table Responses Result in Declining Emission‐Side GHGE

The database was dominated by boreal and temperate peatlands (Figure 1a), and 11,717 CH_4_ and 5252 *R*_eco_ observations with a resolved dominance class fell within the −80 to +30 cm analysis domain (Figure 1b,c). Across the peatland dataset, CH_4_ and *R*_eco_ showed distinct nonlinear responses to WTL (Figure 2). For CH_4_, a two‐breakpoint piecewise model was selected (*n* = 12,305 observations from 242 sites), improving AIC by 129.3 relative to the linear model and by 22.8 relative to the one‐breakpoint model (Figure S1). The breakpoints occurred at −25.4 cm (profile‐likelihood support −31.7 to −22.7 cm) and −8.4 cm (−10.4 to −6.4 cm). Below the first breakpoint, CH_4_ showed little change with WTL (*β*_1_ = 0.001, 95% CI −0.001 to 0.003). CH_4_ increased most strongly between the two breakpoints (*β*_2_ = 0.024, 0.021 to 0.026) and continued to increase above the second breakpoint, but with a smaller slope (*β*_3_ = 0.007, 0.004 to 0.010; Figure 2a). *R*_eco_ followed a simpler one‐breakpoint response (*n* = 5530 observations from 158 sites), with the breakpoint at −26.5 cm (profile‐likelihood support −29.0 to −23.0 cm) and an AIC improvement of 47.9 relative to the linear model. *R*_eco_ declined as WTL rose on both sides of the breakpoint, but the decline was stronger above −26.5 cm (*β*_1_ = −0.012, 95% CI −0.015 to −0.009; *β*_2_ = −0.030, −0.033 to −0.027; Figure 2b). Across the flux–WTL models, marginal *R* ^2^ values were low (0.030–0.081), whereas conditional *R* ^2^ values ranged from 0.33 to 0.61. This indicates that the fixed‐effect hydrological response accounted for only a small proportion of the total variation, with substantial additional variation among sites. The selected structure, breakpoint positions and segment slopes for every gas and vegetation class are given in Table S1.

**FIGURE 2:**
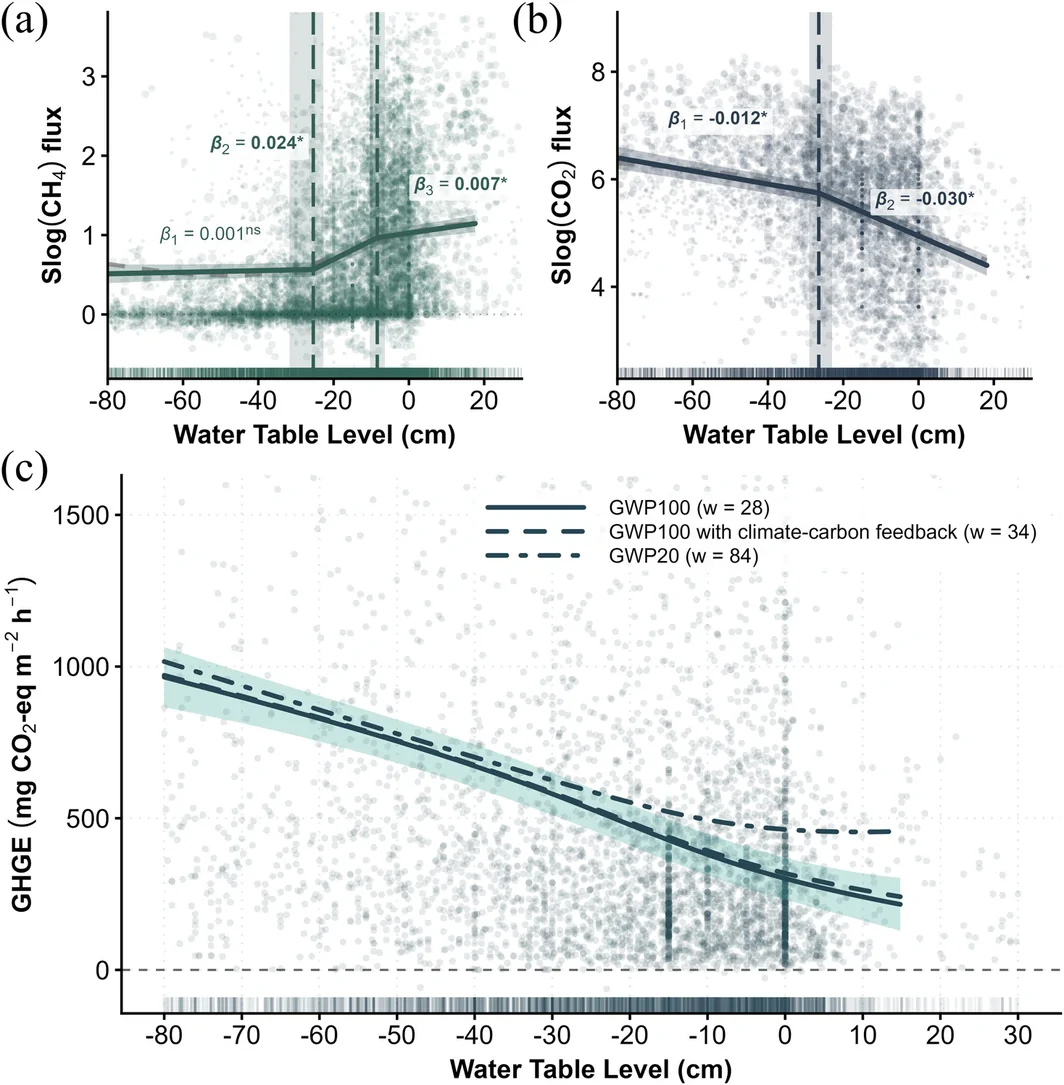
Global water‐table–flux relationships and emission‐side GHGE response in peatlands. (a) CH_4_ flux against water‐table level (WTL) across peat observations within the −80 to +30 cm analysis domain (*n* = 12,305 from 242 sites). A two‐breakpoint piecewise linear mixed model was selected under the ΔAIC > 10 criterion (ΔAIC = 129.3 against the linear model and 22.8 against the one‐breakpoint model), with breakpoints at −25.4 cm (profile‐likelihood support −31.7 to −22.7 cm) and −8.4 cm (−10.4 to −6.4 cm). (b) Ecosystem respiration (*R*_eco_) flux against WTL (*n* = 5530 from 158 sites). A one‐breakpoint model was selected (ΔAIC = 47.9 against the linear model), with a breakpoint at −26.5 cm (−29.0 to −23.0 cm). (c) Emission‐side GHGE against WTL (*n* = 4556 paired CH_4_–*R*_eco_ observations from 120 sites). GHGE was calculated directly from paired observations as *R*_eco_ + *w* × CH_4_ in CO_2_‐equivalent units (mg CO_2_‐eq m^−2^ h^−1^). The primary analysis uses *w* = 28, the 100‐year global warming potential (GWP_100_) for biogenic CH_4_ used throughout the manuscript (solid line). Two alternative IPCC AR5 weights are also shown: *W* = 34 for the 100‐year horizon including climate–carbon feedbacks (dashed line) and *w* = 84 for the 20‐year horizon (dot‐dash line). Observations, rug marks and the 95% confidence band in (c) are shown for *w* = 28 only. In (a, b) the solid line shows the selected model fit and the grey dashed line shows a generalised additive mixed model (GAMM) smooth of the same data for comparison. In (a, b) vertical long‐dashed lines mark the fitted breakpoints, and point area scales with the number of spatial replicates behind each observation. Printed *β*_1_–*β*_3_ are segment slopes (signed‐log units per cm), numbered from the driest segment. Asterisks indicate segment slopes whose 95% confidence intervals exclude zero, and ‘ns’ indicates slopes whose intervals include zero. In (c) curves show GAMM fits for each CH_4_ weighting. In (a, b) shaded bands around breakpoints are profile‐likelihood support intervals. In all panels, shaded ribbons around fitted curves are 95% confidence intervals. Bottom rug marks show data density along the WTL axis. Fitted curves are shown only to the 99th percentile of the WTL distribution in each panel (a) +17.7 cm; (b) +18.1 cm; (c) +15.0 cm to avoid extrapolation. All models include site as a random intercept and are weighted by ln(*N*) + 1, where N is the number of spatial replicates represented by each observation. Fluxes in (a, b) (mg CH_4_ m^−2^ h^−1^ and mg CO_2_ m^−2^ h^−1^) are signed‐log transformed as slog(*x*) = sign(*x*) × ln(1 + |*x*|).

Combining paired CH_4_ and *R*_eco_ observations yielded an emission‐side GHGE response that declined towards wetter conditions under the primary CH_4_ weighting of *w* = 28 (*n* = 4556 paired observations from 120 sites, Figure 2c). No internal minimum was identified within the analysed WTL range. We estimated the change in GHGE between WTL = −60 cm, which was close to the fifth percentile of the paired observations, and WTL = +15 cm, which corresponded to the 99th percentile and was used as the upper limit of the fitted curve. GHGE declined by 73.9%, from 827.4 to 216.0 mg CO_2_‐eq m^−2^ h^−1^. Across the same WTL range, the decline was 71.0% under *w* = 34 and 46.6% under *w* = 84. The magnitude of the decline above the peat surface depended strongly on CH_4_ weighting. From WTL = 0 to +15 cm, GHGE declined by 28.4% under *w* = 28, 24.4% under *w* = 34 and 1.4% under *w* = 84. The dependence of this decline on the CH_4_ weighting is shown across a continuous range of weights in Figure S2.

### 3.2 Flux–Water‐Table Responses Differ Between Dominant Vegetation Classes

Flux–WTL relationships differed between dominant vegetation classes for both gases (Figure 3). For CH_4_ (*n* = 11,717 observations from 227 sites), adding vegetation class improved model fit (χ^2^ = 31.75, *p* = 1.75 × 10^−8^), and the vegetation × WTL interaction provided further improvement (χ^2^ = 24.48, *p* = 7.50 × 10^−7^). For *R*_eco_ (*n* = 5252 observations from 147 sites), there was no detectable overall difference in flux level between vegetation classes (χ^2^ = 0.06, *p* = 0.811), but the vegetation × WTL interaction was significant (χ^2^ = 12.33, *p* = 4.46 × 10^−4^). We therefore examined the hydrological responses separately for GRA and NGRA peatlands. A three‐state classification, which separates NGRA observations with graminoids present from those without, provided a better fit to the flux–WTL interaction than the binary dominance classification (CH_4_ χ^2^ = 48.9, *p* = 2.7 × 10^−12^, *n* = 11,537 from 222 sites; *R*_eco_ χ^2^ = 7.6, *p* = 0.006, *n* = 5115 from 142 sites; Table S2). We retain the dominance classification in Figures 3 and 4 because that is the resolution at which vegetation is represented in land‐surface models, and Figures S3 and S7 show the additional variation captured by graminoid presence.

**FIGURE 3:**
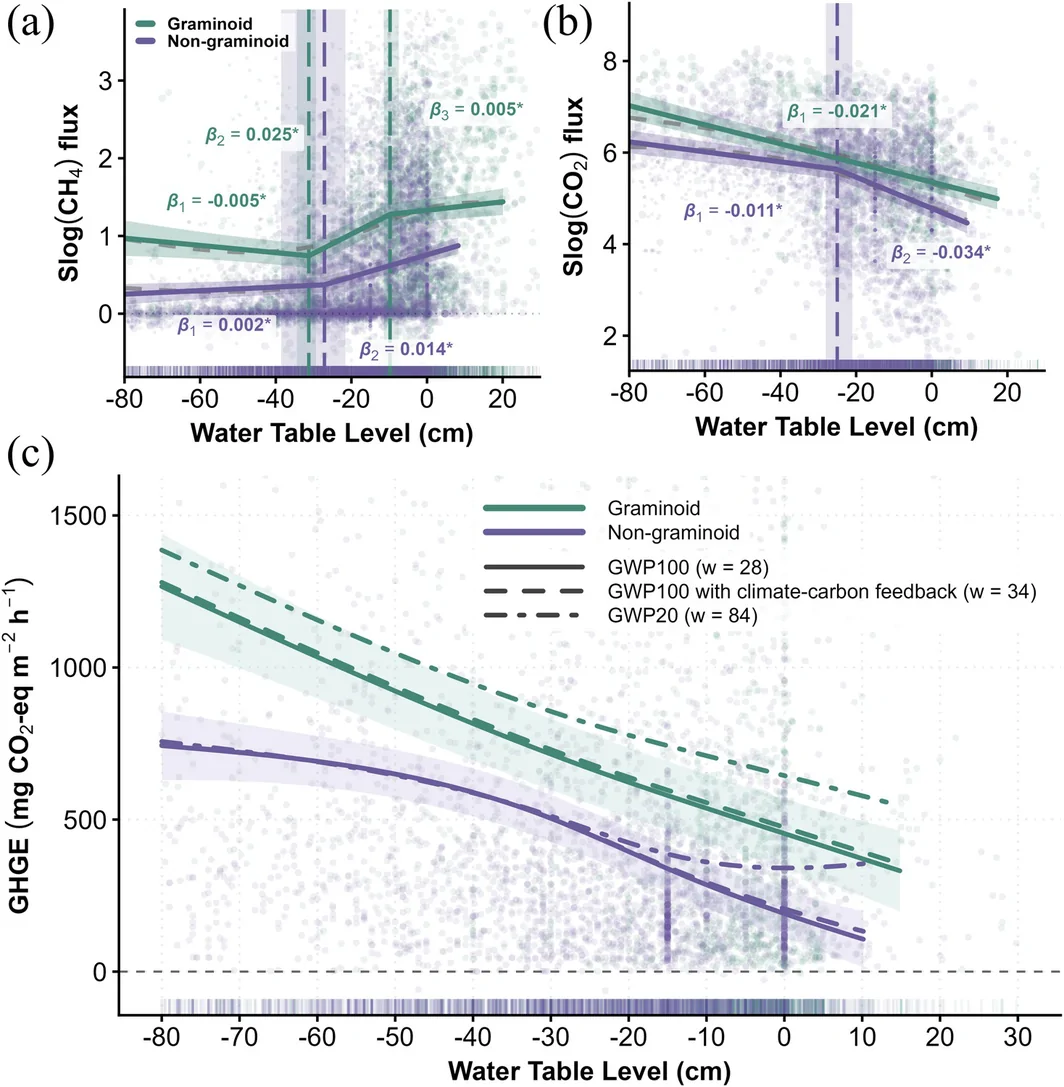
Flux–water‐table relationships stratified by vegetation class. (a) CH_4_ flux against water‐table level (WTL) in graminoid‐dominated (GRA, green; *n* = 4283 from 113 sites) and non‐graminoid‐dominated (NGRA, purple; *n* = 7434 from 132 sites) peatlands. A two‐breakpoint model was selected for GRA, with breakpoints at −31.3 cm (profile‐likelihood support −34.4 to −29.4 cm) and −9.8 cm (main support interval −11.4 to −7.4 cm), and segment slopes of *β*_1_ = −0.005, *β*_2_ = 0.025 and *β*_3_ = 0.005. Profile‐likelihood support for the second GRA CH_4_ breakpoint was disjoint. A one‐breakpoint model was selected for NGRA, with a breakpoint at −27.1 cm (−38.6 to −21.6 cm) and slopes of *β*_1_ = 0.002 and *β*_2_ = 0.014. (b) Ecosystem respiration (*R*_eco_) against WTL by vegetation class (GRA: *N* = 1662 from 49 sites; NGRA: *N* = 3590 from 108 sites). A linear model was retained for GRA (*β*_1_ = −0.021) because the one‐breakpoint model improved AIC by only 9.1, below the prespecified ΔAIC > 10 threshold. A one‐breakpoint model was selected for NGRA, with a breakpoint at −25.0 cm (−28.0 to −21.0 cm) and slopes of *β*_1_ = −0.011 and *β*_2_ = −0.034. In (a, b) the coloured solid line shows the selected model fit. (c) Emission‐side GHGE against WTL by vegetation class (GRA: *N* = 1517 from 44 sites; NGRA: *N* = 2838 from 76 sites). GHGE was calculated directly from paired CH_4_–*R*_eco_ observations as *R*_eco_ + *w* × CH_4_ in CO_2_‐equivalent units. Solid, dashed and dot‐dash lines show *w* = 28 (GWP_100_), *w* = 34 (GWP_100_ including climate–carbon feedbacks) and *w* = 84 (GWP_20_), respectively. Observations and 95% confidence bands are shown for *w* = 28 only. Fitted curves are shown only to the 99th percentile of each class's own WTL distribution (a, +20.2 cm for GRA and +8.5 cm for NGRA; b, +17.4 cm and +9.4 cm; c, +15.0 cm and +10.4 cm). Other plotting conventions follow Figure 2.

**FIGURE 4:**
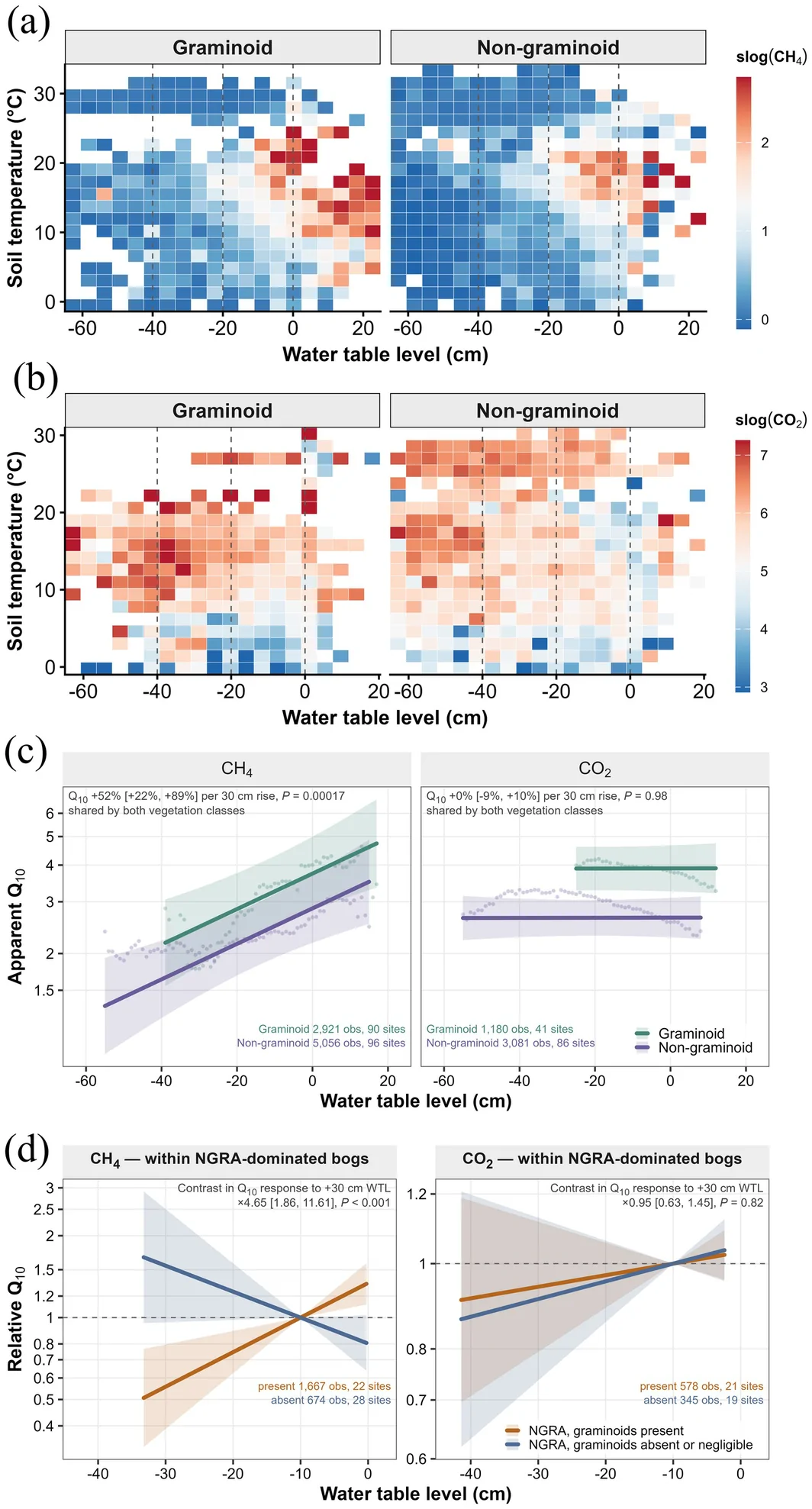
Hydrology‐modulated temperature sensitivity. (a, b) Flux intensity across the water‐table level (WTL) × soil‐temperature space, shown separately for graminoid‐dominated (GRA) and non‐graminoid‐dominated (NGRA) peatlands. (a) CH_4_ flux (GRA: *N* = 3195 from 90 sites; NGRA: *N* = 6332 from 99 sites). (b) Ecosystem respiration (*R*_eco_) flux (GRA: *N* = 1146 from 41 sites; NGRA: *N* = 2943 from 86 sites). Colour shows mean signed‐log‐transformed flux (as in Figure 2) within each WTL × soil‐temperature bin. Grey dashed vertical lines at WTL = −40, −20 and 0 cm are visual guides and do not mark breakpoints. (c) Apparent Q_10_ against WTL from the hierarchical varying‐coefficient model. Solid lines and shaded ribbons show fitted values and 95% confidence intervals for each dominant vegetation class. The selected model allows vegetation‐specific Q_10_ levels but uses a shared WTL modulation within each gas. Printed values give this shared change in apparent Q_10_ per 30 cm rise in WTL, with its 95% confidence interval and *p* value. Faint points show the 30‐cm sliding‐window within‐site estimates, which are included for comparison only, because neighbouring windows share observations and are not independent. Curves are shown only over class‐specific supported ranges where at least 20 sites can provide an informative within‐site temperature slope (CH_4_: GRA −39 to +17 cm, NGRA −55 to +15 cm; *R*_eco_: GRA −25 to +12 cm, NGRA −55 to +8 cm). Sample sizes in (c) differ from those in (a, b) because Q_10_ estimation on log‐transformed fluxes uses only positive fluxes, taken from the −80 to +30 cm WTL domain (CH_4_: GRA *n* = 2921 from 90 sites, NGRA *n* = 5056 from 96 sites; *R*_eco_: GRA *n* = 1180 from 41 sites, NGRA *n* = 3081 from 86 sites), whereas the flux‐intensity maps in a and b include all fluxes between −65 and +25 cm. Flexible and linear WTL‐modulation fits are compared in Figure S4, and site support and its threshold sensitivity are shown in Figure S5 and Table S5. (d) Relative Q_10_ against WTL within NGRA bogs, separated by graminoid presence, for CH_4_ (left; graminoids present: *N* = 1667 from 22 sites; absent or negligible: *N* = 674 from 28 sites) and *R*_eco_ (right; present: *N* = 578 from 21 sites; absent or negligible: *N* = 345 from 19 sites). Curves are normalised to 1 at the display reference of WTL = −10 cm so that the panels compare WTL modulation rather than absolute Q_10_. Each flux is shown over the common WTL support of its two groups (CH_4_ −33 to 0 cm; *R*_eco_ −41 to −2 cm). Shaded bands are 95% confidence intervals, which have zero width at −10 cm because the curves are normalised there. The grey dashed line marks a relative Q_10_ of 1. The printed contrast is the ratio of the 30 cm Q_10_ multipliers between the graminoid‐present and graminoid‐absent or negligible groups, with its 95% confidence interval and *p* value. Sensitivity analyses for the within‐bog comparison are reported in Table S4.

For CH_4_, a two‐breakpoint model was selected for GRA peatlands (*n* = 4283 observations from 113 sites), with breakpoints at −31.3 cm (profile‐likelihood support −34.4 to −29.4 cm) and −9.8 cm (main profile‐likelihood support −11.4 to −7.4 cm; Figure 3a). CH_4_ declined slightly below the first breakpoint (*β*_1_ = −0.005), increased strongly between the two breakpoints (*β*_2_ = 0.025), and continued to increase above −9.8 cm with a smaller slope (*β*_3_ = 0.005). In NGRA peatlands (*n* = 7434 observations from 132 sites), a one‐breakpoint model was selected, with the breakpoint at −27.1 cm (−38.6 to −21.6 cm). CH_4_ increased weakly below the breakpoint (*β*_1_ = 0.002) and more strongly above it (*β*_2_ = 0.014).

*R*_eco_ showed a different vegetation contrast (Figure 3b). In GRA peatlands (*n* = 1662 observations from 49 sites), the linear model was retained under the prespecified selection rule. Adding a breakpoint reduced AIC by 9.1 units, which was below the required improvement of 10. The fitted *R*_eco_ response therefore declined linearly with rising WTL on the signed‐log scale (*β*_1_ = −0.021). In NGRA peatlands (*n* = 3590 observations from 108 sites), a one‐breakpoint model was selected at −25.0 cm (profile‐likelihood support −28.0 to −21.0 cm). *R*_eco_ declined more slowly below this breakpoint (*β*_1_ = −0.011) and more strongly above it (*β*_2_ = −0.034).

Emission‐side GHGE also showed different responses to WTL between vegetation classes (Figure 3c). In GRA peatlands (*n* = 1517 paired observations from 44 sites), fitted GHGE declined from WTL = 0 to +15 cm by 27.0% under *w* = 28, 25.3% under *w* = 34, and 15.4% under *w* = 84. In NGRA peatlands (*n* = 2838 paired observations from 76 sites), where the fitted curves extend to +10.4 cm, GHGE declined from WTL = 0 to +10.4 cm by 44.4% under *w* = 28 and by 36.0% under *w* = 34 but increased slightly by 4.1% under *w* = 84. Thus, above the peat surface, GHGE declined under all three weightings in GRA peatlands, whereas in NGRA peatlands the response changed direction under *w* = 84.

### 3.3 Apparent CH_4_ Temperature Sensitivity Increases With WTL

Flux patterns across the WTL × soil‐temperature space differed between CH_4_ and *R*_eco_ (Figure 4a,b). CH_4_ fluxes were generally low under dry conditions, while temperature‐associated variation became more pronounced as WTL approached the peat surface. *R*_eco_ showed a temperature gradient across a broader WTL range. The hierarchical within‐site models quantified the WTL dependence of apparent Q_10_ (Figure 4c). For CH_4_, apparent Q_10_ increased by a factor of 1.149 per 10 cm rise in WTL (95% CI 1.069–1.235), equivalent to a factor of 1.517 over a 30 cm rise (95% CI 1.221–1.886, *p* = 1.7 × 10^−4^). Wetter conditions were therefore associated with higher CH_4_ temperature sensitivity. The within‐site temperature × WTL interaction remained positive when all 9772 eligible CH_4_ observations from 176 sites, including zero and negative fluxes, were analysed on the signed‐log scale (coefficient 0.0014, 95% CI 0.0012–0.0016, *p* = 4.6 × 10^−35^). No corresponding WTL dependence was detected for *R*_eco_, for which the 30 cm Q_10_ ratio was 1.001 (95% CI 0.913–1.098, *p* = 0.975). The same space partitioned by graminoid presence rather than by dominance is shown in Figure S6.

We next tested whether hydrological modulation differed between dominant vegetation classes. The model with vegetation‐specific Q_10_ levels and shared WTL modulation had the lowest AIC for both gases (REDUCED). Allowing the modulation to differ between classes increased AIC by 1.87 for CH_4_ and 0.95 for *R*_eco_ (FULL). These small differences did not clearly distinguish shared from vegetation‐specific modulation. We therefore retained the shared modulation as the simpler summary. Compared with a common Q_10_ level across classes (COMMON), vegetation‐specific levels reduced AIC by 21.72 for *R*_eco_ and 2.57 for CH_4_. However, the estimated difference in apparent *R*_eco_ Q_10_ between vegetation classes was sensitive to site grouping (Table S6). Separate smooth WTL responses fitted within each gas × vegetation group reduced AIC by at most 2.64 units in three combinations and increased AIC in the fourth (Figure S4). At the whole‐dataset scale, the three‐state classification did not improve on either binary classification for CH_4_ Q_10_ modulation (*p* = 0.057 against the dominance classification and *p* = 0.24 against the presence classification; Table S2). Within bogs, where wetland type is held constant, the three‐state classification was supported over both binary classifications (χ^2^ = 15.9, *p* = 6.8 × 10^−5^ against the dominance classification; χ^2^ = 4.0, *p* = 0.046 against the presence classification). In this all‐bog model, the WTL modulation of CH_4_ Q_10_ was 8.96 times larger (95% CI 3.43–23.40, *p* = 7.5 × 10^−6^) where graminoids were present than where they were absent (Figure S7). The wetland‐type composition of the three states is given in Table S3.

We then tested whether finer vegetation information separated the responses within NGRA bogs (Figure 4d). For CH_4_, the graminoid‐present group contained 1667 observations from 22 sites, while the graminoid‐absent or negligible group contained 674 observations from 28 sites. The ratio of the 30 cm Q_10_ multipliers between the graminoid‐present and graminoid‐absent or negligible groups was 4.65 (95% CI 1.86–11.61, *p* = 0.001). The fitted CH_4_ Q_10_ responses ran in opposite directions, increasing towards wetter conditions when graminoids were present and decreasing when they were absent or negligible. For *R*_eco_, the corresponding groups contained 578 observations from 21 sites and 345 observations from 19 sites. No corresponding difference was detected (ratio = 0.95, 95% CI 0.63–1.45, *p* = 0.82). The CH_4_ contrast was retained in sensitivity analyses that treated peatland complexes as the clustering unit (Table S4).

### 3.4 A Hydrological Q_10_ Scaling for Model Implementation

We translated the shared hydrological modulation into a simple Q_10_ scaling for model implementation (Figure 5). The CH_4_ model included 7977 observations from 173 sites. Its scaling was evaluated over the supported WTL range of −63 to +20 cm. A 10 cm rise in WTL corresponded to a 1.149‐fold change in Q_10_ (Table 1). The within‐site temperature coefficient varied approximately linearly with WTL. Allowing this coefficient to vary smoothly did not improve model fit (effective degrees of freedom = 2.0, ΔAIC = +0.05). We therefore retained a constant 1.149‐fold change in Q_10_ per 10 cm rise in WTL.

**FIGURE 5:**
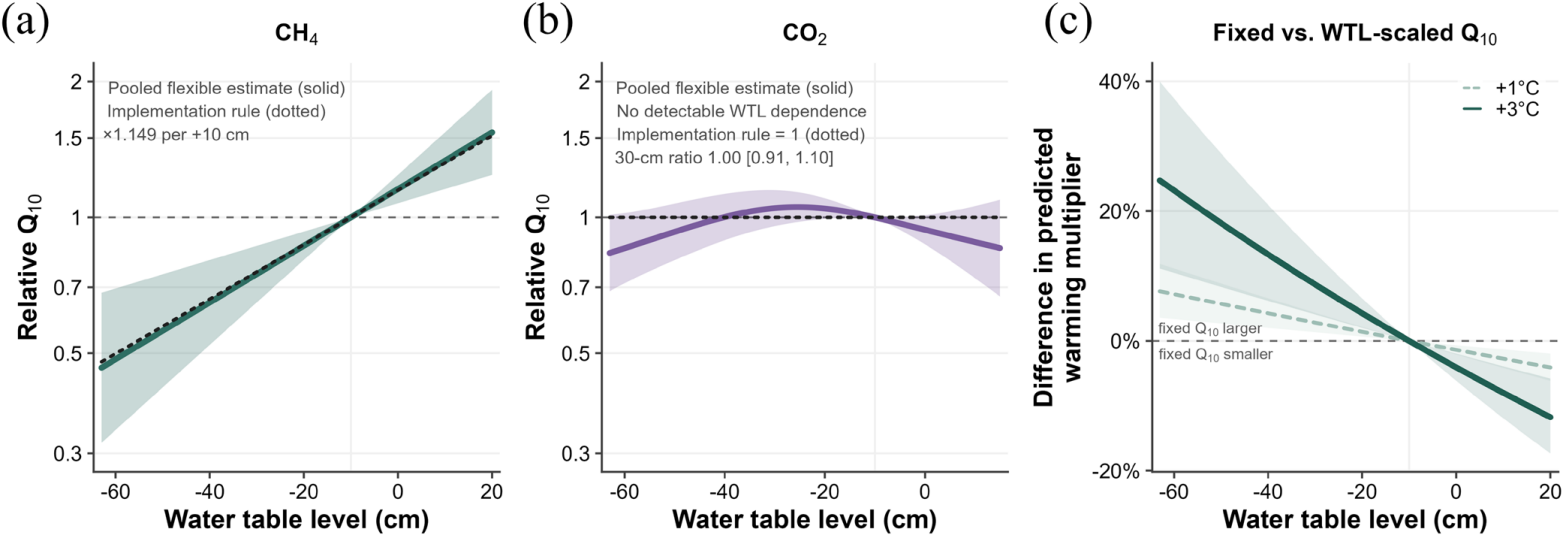
Shared hydrological scaling of apparent Q_10_ for model implementation. Panels (a,b) show the relative multiplier *R*(WTL) = Q_10_ (WTL)/Q_10_ (−10 cm). For the implementation rules, this normalisation removes the vegetation‐specific Q_10_ level and leaves the WTL modulation shared across vegetation classes as a single curve. The dotted lines show the implementation rules, which follow the shared linear WTL modulation of the model in Table 1. Figure 4c shows the same model on the absolute Q_10_ scale, where the vegetation‐specific levels appear as separate curves. The solid lines and ribbons show a separate flexible fit in which Q_10_ is pooled across vegetation classes. This fit was used to check the linear form. (a) Relative CH_4_ Q_10_ against water‐table level (WTL), normalised to 1 at the display reference of WTL = −10 cm (*n* = 7977 from 173 sites). The solid line and shaded ribbon show the flexible estimate and its 95% confidence interval. The dotted line shows the implementation rule of ×1.149 per 10 cm rise in WTL (Table 1). (b) Relative *R*_eco_ Q_10_ against WTL (*n* = 4261 from 119 sites), shown using the same scale as (a) The solid line and ribbon show the flexible estimate and 95% confidence interval. The dotted horizontal line represents the implementation rule of no hydrological correction. The printed 30‐cm ratio is the *R*_eco_ Q_10_ ratio for a 30 cm rise in WTL from the linear model, with its 95% confidence interval (Table 1). (c) Relative difference in the predicted CH_4_ warming multiplier when Q_10_ is held fixed at its value at WTL = −10 cm, calculated as ${\left(Q_{10}\left(-10\right)/Q_{10}\left(\mathrm{WTL}\right)\right)}^{\Delta T/10}-1$ and expressed as a percentage. The +1°C response is shown by the thinner short‐dashed line and the +3°C response by the thicker solid line. Positive values indicate that the fixed‐Q_10_ formulation gives a larger multiplier than the WTL‐dependent formulation, and negative values indicate a smaller multiplier. A grey dashed horizontal line marks no change: *R* = 1 in (a, b) and zero difference in (c) Shaded bands in c propagate uncertainty in the CH_4_ scaling coefficient, conditional on the linear implementation rule. Curves are shown only over WTL ranges where at least 20 informative sites are available across the combined vegetation subset (CH_4_ −63 to +20 cm; *R*_eco_ − 63 to +15 cm). These ranges were obtained using the same support criterion as the vegetation‐specific estimates. Class‐specific support and its threshold sensitivity are shown in Figure S5 and Table S5. WTL = −10 cm does not represent a hydrological threshold. The absolute implementation in Table 1 is anchored at WTL = 0 cm.

**TABLE 1:** Water‐table scaling of apparent Q_10_.

Flux	Scaling of Q_10_ per + 10 cm WTL	Effect over a 30 cm rise	*p*	Interpretation
CH_4_	1.149 (95% CI 1.069–1.235)	1.517 (95% CI 1.221–1.886)	1.7 × 10^−4^	WTL‐dependent
*R*_eco_	1.000 (95% CI 0.970–1.032)	1.001 (95% CI 0.913–1.098)	0.975	No detectable WTL dependence

*Note:* The scaling was estimated from the hierarchical varying‐coefficient model of Section 2.2.2 with the modulation shared across vegetation classes. Estimates are based on 7977 positive CH_4_ flux observations from 173 sites and 4261 positive *R*_eco_ observations from 119 sites, and apply over the supported WTL ranges of −63 to +20 cm for CH_4_ and −63 to +15 cm for *R*_eco_ (Figure 5). The scaling is applied as Q_10_(WTL) = Q_10_(0) × *r* ^(WTL/10)^, where *r* is the multiplicative change in apparent Q_10_ per 10 cm rise in WTL given in the table, and Q_10_(0) is the apparent Q_10_ at the peat surface (WTL = 0 cm). For *R*_eco_, no hydrological correction is applied (*r* = 1) because no WTL dependence was detected. *p* values are from Wald tests of whether *r* differs from 1. Absolute Q_10_(0) values by vegetation class are given in Table S6.

The *R*_eco_ model included 4261 observations from 119 sites, with a supported WTL range of −63 to +15 cm (Figure 5b). No detectable WTL dependence of Q_10_ was found (Table 1). A flexible WTL response reduced AIC by 2.85 units relative to the linear form, providing limited support for additional hydrological structure. We therefore included no hydrological correction to Q_10_ in the simplified *R*_eco_ implementation. We then compared warming responses estimated using fixed and WTL‐dependent CH_4_ Q_10_ values (Figure 5c). The fixed Q_10_ was set to the estimated value at WTL = −10 cm. Relative differences in the predicted flux multiplier were calculated using the WTL‐dependent estimate as the reference. Under +3°C warming, the fixed‐Q_10_ multiplier was 24.7% higher at WTL = −63 cm and 11.8% lower at +20 cm. These differences were larger in magnitude under +3°C than under +1°C warming.

## 4 Discussion

### 4.1 Emission‐Side GHGE Declines With Rising WTL, but the Above‐Surface Response Depends on CH_4_ Weighting

Across our dataset, emission‐side GHGE declined as WTL rose under the primary CH_4_ weighting of *w* = 28, with no internal minimum within the analysed range (Figure 2c). The decline from deeply drained to wetter conditions persisted across all three CH_4_ weightings. However, the emission‐side benefits of further inundation were less consistent: there was a small overall decline under *w* = 84, while GHGE increased slightly in NGRA peatlands under this weighting over their supported above‐surface range (Figure 3c). Previous studies have identified low‐emission conditions near or below the peat surface (Evans et al. 2021; Koch et al. 2023; Zou et al. 2022). However, differences in flux components and accounting frameworks limit direct comparison. Our metric includes only *R*_eco_ and CH_4_, so it cannot establish an optimal WTL for the full ecosystem GHG balance. Assessing the climate benefits of rewetting requires comparing the time‐dependent effects of avoided CO_2_ emissions and additional CH_4_ emissions against continued drainage (Günther et al. 2020).

### 4.2 Vegetation Classes Differ in CH_4_ and *R*_eco_ Responses to WTL

*R*_eco_ declined continuously in both vegetation classes as WTL rose (Figure 3b). This pattern is consistent with reduced aerobic decomposition as peat becomes wetter and oxygen availability declines (Bridgham et al. 2013). The vegetation contrast concerned the shape rather than the direction of this decline. In a peat mesocosm experiment, sedge treatments with lowered water tables had the greatest proportional contribution of peat carbon to ecosystem respiration (Stuart et al. 2023). Differences in the contributions of plant and peat respiration may therefore help explain the contrasting *R*_eco_ responses across vegetation classes. However, because *R*_eco_ integrates both autotrophic and heterotrophic components, our data cannot determine which component accounts for the vegetation contrast.

GRA peatlands showed a steeper principal increase in CH_4_ on the transformed flux scale, while the first breakpoint support intervals overlapped between vegetation classes (Figure 3a). Substrate supply from graminoid root exudates (Bhullar et al. 2014; Ström et al. 2003) and CH_4_ transport through aerenchyma (Joabsson et al. 1999; Whiting and Chanton 1992) could both contribute to the higher CH_4_ emissions observed in GRA peatlands. Root oxygen release can act in the opposite direction by promoting CH_4_ oxidation in the rhizosphere (Armstrong and Armstrong 2005; Laanbroek 2010), although barriers to radial oxygen loss may restrict this effect to particular parts of the root system (Colmer 2003). These processes could help explain the steeper CH_4_–WTL response if their relative importance changes with WTL. This mechanistic interpretation could not be tested directly with our dataset. The steeper CH_4_ increase in GRA peatlands is relevant to restoration because rewetted fens can develop tall graminoid communities that remain distinct from near‐natural vegetation for decades (Kreyling et al. 2021).

The broad NGRA category includes Sphagnum‐, shrub‐ and forest‐dominated peatlands, where CH_4_ transport pathways and microbial controls may differ. NGRA classification also does not imply that graminoids are absent. Where plant‐mediated transport is limited, CH_4_ moving by diffusion may undergo substantial oxidation in the oxic peat layer, although ebullition provides another release pathway (Bridgham et al. 2013). In Sphagnum‐dominated peatlands, moss‐associated methanotrophs can oxidise CH_4_ within the moss layer (Larmola et al. 2010; Raghoebarsing et al. 2005). Acidic pore waters may also inhibit acetoclastic methanogenesis (Bridgham et al. 2013). These processes could contribute to lower CH_4_ emissions in some Sphagnum‐dominated systems, but their importance may differ in shrub‐ and forest‐dominated peatlands. Our results are consistent with earlier evidence that vegetation influences CH_4_ responses to WTL (Bao et al. 2021), but do not identify which processes account for the contrast. The three‐state classification also provided a better fit than the dominance classification in several analyses (Table S2). This suggests that graminoid presence may capture variation that is not represented by dominant vegetation alone.

### 4.3 WTL Dependence of Apparent CH_4_ Q_10_

Apparent CH_4_ Q_10_ increased towards wetter conditions. A shared WTL modulation adequately described this increase across GRA and NGRA peatlands, although this does not establish identical responses between the two classes. Riutta et al. (2020) showed that water level conditioned the influence of vegetation on CH_4_ flux in a boreal fen. Our synthesis adds a distinction between the hydrological responses of flux magnitude and temperature sensitivity. CH_4_ flux showed changes in slope along the WTL gradient (Figure 3a), whereas apparent CH_4_ Q_10_ increased continuously and was adequately represented by a constant multiplicative change per 10 cm rise in WTL (Figure 5a). In a supplementary comparison within NGRA bogs, fitted CH_4_ Q_10_ increased with WTL where graminoids were present but decreased where they were absent or negligible (Figure 4d). No corresponding contrast was detected for *R*_eco_. This CH_4_ contrast was robust to clustering by peatland complex and omission of individual complexes (Table S4). This suggests that graminoids may influence CH_4_ temperature responses even when they do not dominate the vegetation. Changes in CH_4_ temperature sensitivity might therefore accompany, or potentially precede, shifts in dominant vegetation, although our cross‐sectional data cannot establish the temporal sequence.

Experimental evidence also shows that vegetation can alter CH_4_ temperature sensitivity: Leroy et al. (2017) found that *Molinia caerulea* increased CH_4_ temperature sensitivity in Sphagnum‐dominated peat mesocosms. Turner et al. (2020) found that vascular vegetation increased CH_4_ production and reduced oxidation in a thermokarst bog. These processes can affect apparent Q_10_ because CH_4_ production and oxidation have different temperature responses and environmental controls (Zhang et al. 2021). Their balance may also shift with WTL (Riutta et al. 2020). Although we did not measure substrate supply or plant‐mediated transport directly, we propose that these processes may help sustain a stronger temperature response of emitted CH_4_ under wetter conditions where graminoids are present. Measurements of root traits, CH_4_ production and oxidation are needed to test this explanation and resolve the contrasting response directions. For *R*_eco_, we found no detectable WTL dependence of apparent Q_10_ despite declining fluxes towards wetter conditions. This suggests that hydrological controls on respiration rates need not produce a corresponding change in apparent temperature sensitivity. Although the model favoured vegetation‐specific Q_10_ levels, this contrast was sensitive to site grouping (Table S6). We therefore regard the vegetation contrast in the level of *R*_eco_ Q_10_ as less robust than the WTL dependence of CH_4_ Q_10_.

### 4.4 Implications for Modelling Peatland Responses to Warming

The hydrological dependence of apparent CH_4_ Q_10_ provides a simple relationship for modelling temperature responses. Under +3°C warming, fixing Q_10_ at its value at WTL = −10 cm gave a warming flux multiplier that was 24.7% higher at −63 cm and 11.8% lower at +20 cm than that obtained using the WTL‐dependent formulation (Figure 5c). These differences depend on the reference WTL chosen for the fixed Q_10_ and describe a model comparison, not independently measured prediction errors. This implementation summarises the relationship across the observation network and requires validation before application to individual sites. A further modelling question is whether the same warming produces different annual CH_4_ responses when concentrated in wet versus dry periods.

Studies of ecosystem and soil respiration have likewise incorporated variation in temperature sensitivity with environmental conditions (Kim et al. 2019; Niu et al. 2021). For CH_4_, Basso et al. (2026) found that no single temperature‐sensitivity parameter setting gave the best agreement with atmospheric constraints across all Arctic subregions. Their parameters describe CH_4_ production within a process model, whereas our apparent Q_10_ describes the temperature response of observed fluxes. The two quantities are therefore not directly interchangeable. Our WTL‐dependent relationship provides an observational benchmark for evaluating whether models reproduce hydrological variation in apparent CH_4_ temperature sensitivity. Agreement with mean CH_4_ emissions alone may therefore conceal errors in the simulated warming response across hydrological conditions. Incorporating hydrological dependence in CH_4_ temperature‐sensitivity parameterisations could improve the representation of warming responses, particularly where models simulate substantial shifts in WTL.

### 4.5 Limitations and Priorities for Future Research

Our dataset is dominated by boreal and temperate peatlands. Further observations from tropical and subtropical peatlands are needed to assess whether these relationships extend to warmer systems with different vegetation, substrate supply, and CH_4_ transport pathways. The GHGE analysis covers *R*_eco_ and CH_4_ emissions but excludes photosynthetic CO_2_ uptake and lateral carbon losses. An exploratory GPP correction, applied as a constant WTL slope derived from the eddy‐covariance GPP models, retained about 81% of the emission‐side WTL response, with corresponding values of 78% in NGRA and 85% in GRA peatlands (Figure S8). The response retained its direction under this correction, but the comparison does not establish the full ecosystem GHG response across the wider dataset. Our synthesis also does not resolve short‐term responses immediately after rewetting or establish how individual sites change through time.

Static chambers provide fine spatial resolution for vegetation comparisons, but sampling may miss episodic ebullition (Strack et al. 2005) and chamber placement may exclude tree‐stem CH_4_ emissions (Covey and Megonigal 2019; Pangala et al. 2017). These limitations may affect how well the observations represent total emissions across vegetation types. The low marginal *R* ^2^ values indicate that the fixed effects explain a limited share of flux variation, so the fitted relationships describe broad patterns rather than precise predictions for individual sites. Vegetation contrasts may partly reflect differences between minerotrophic fens and ombrotrophic bogs, which are more common in GRA and NGRA sites, respectively. Restricting the comparison to bogs reduces this confounding but does not isolate vegetation effects from other site conditions. Future datasets that combine quantitative vegetation cover, plant traits and site conditions would help distinguish these influences.

## 5 Conclusion

Understanding peatland responses to rewetting and warming requires linking hydrological controls on emission rates with hydrological controls on temperature sensitivity. Our synthesis shows that emission‐side GHGE declined as WTL rose under all three CH_4_ weightings, but that the response to further inundation depended on CH_4_ weighting and vegetation class. CH_4_ and *R*_eco_ responses to WTL differ between dominant vegetation classes, while wetter conditions are associated with greater apparent CH_4_ Q_10_. The WTL‐dependent Q_10_ relationship provides an observational benchmark for testing whether models capture this hydrological variation. Independent observations are needed to assess its predictive value across peatland types and under different climatic conditions. Representing vegetation differences in flux–WTL responses and the WTL dependence of CH_4_ Q_10_ in models that account for the full ecosystem carbon balance could help evaluate how rewetting influences peatland climate forcing under future warming. Such modelling could also inform long‐term restoration planning.

## Author Contributions

**Heyu Chen:** conceptualization, methodology, data curation, writing – original draft, formal analysis, visualization, software, investigation, writing – review and editing, funding acquisition, project administration. **Jirateep Yotmaw:** data curation, visualization, writing – review and editing. **Liam Thompson:** visualization, writing – review and editing. **Dominik Bittner:** visualization, writing – review and editing, software. **Yongxing Ren:** visualization, writing – review and editing, data curation, software, funding acquisition, resources. **Yi Xu:** writing – review and editing, visualization. **Scott J. Davidson:** writing – review and editing, resources. **Jingui Wei:** writing – review and editing. **Wen Yin:** writing – review and editing, funding acquisition, software, resources. **Grant Campbell:** writing – review and editing. **Shuai Xue:** writing – review and editing. **Chris D. Evans:** writing – review and editing, validation, methodology, conceptualization. **Pete Smith:** writing – review and editing, conceptualization, methodology. **Astley Hastings:** supervision, writing – review and editing, validation, methodology, conceptualization. **Mohamed Abdalla:** methodology, validation, writing – review and editing, supervision, conceptualization.

## Funding

This work was supported by the National Natural Science Foundation of China (grant no. 42494821), the Science and Technology Projects of Xizang Autonomous Region, China (grant no. XZ202601ZY0195) and the Scientific Research Innovation Capability Support Project for Young Faculty (grant no. SRICSPYF‐BS2025119). H.C. was additionally supported by the J.W. Stephen Fellowship for international collaboration, School of Biological Sciences, University of Aberdeen.

## Conflicts of Interest

The authors declare no conflicts of interest.

## Supporting information


**Figure S1:** AIC comparison of candidate flux–WTL models.
**Figure S2:** Dependence of wet‐end GHGE change on CH_4_ weighting.
**Figure S3:** Flux and emission‐side GHGE by graminoid presence.
**Figure S4:** Comparison of sliding‐window and hierarchical estimates of apparent Q_10_.
**Figure S5:** Site support for within‐site temperature slopes across the water‐table gradient.
**Figure S6:** Flux and apparent Q_10_ across the water‐table–temperature space, grouped by graminoid presence.
**Figure S7:** Water‐table modulation of apparent CH_4_ Q_10_ under a three‐state vegetation classification.
**Figure S8:** Effect of a GPP correction on the water‐table response of emission‐side GHGE.
**Figure S9:** Breakpoint structure and location under three weighting schemes.
**Figure S10:** Sliding‐window apparent Q_10_ profiles.
**Figure S11:** Funnel plot of site‐level CH_4_ sensitivity estimates.
**Table S1:** Selected structural models for the flux–water‐table relationships.
**Table S2:** Comparison of binary and three‐state vegetation classifications for the WTL interaction.
**Table S3:** Wetland‐type composition of the three vegetation states.
**Table S4:** Robustness of the within‐bog graminoid‐presence contrast in CH_4_ Q_10_ modulation.
**Table S5:** Supported water‐table ranges under three informative‐site thresholds.
**Table S6:** Apparent Q_10_ at the peat surface by gas and vegetation class.
**Table S7:** Breakpoint structure under four treatments of extreme fluxes.
**Table S8:** Eddy‐covariance sites used for the GPP–WTL sensitivity analysis.
**Table S9:** Site‐by‐site vegetation curation.

## Data Availability

The data and code that support the findings of this study are available on Zenodo at https://doi.org/10.5281/zenodo.22727180. The chamber flux database was compiled from published studies and from the global wetland CO_2_–CH_4_ flux database of Chen et al. (2021; https://doi.org/10.5281/zenodo.5113602), ABCFlux v2 (https://doi.org/10.3334/ORNLDAAC/2448), the Arctic Data Center (https://arcticdata.io), PANGAEA (https://www.pangaea.de) and the UKCEH Environmental Information Data Centre (https://catalogue.ceh.ac.uk). The eddy‐covariance data used for the GPP–WTL sensitivity analysis were obtained from AmeriFlux (https://ameriflux.lbl.gov), the FLUXNET‐CH4 Community Product (https://fluxnet.org/data/fluxnet‐ch4‐community‐product/), the UK‐SCAPE peatland tower network (https://catalogue.ceh.ac.uk), the Netherlands peatland eddy‐covariance network and the original publications listed in Table S8.
