# Supplementary material for: Water Table and Vegetation Shape Peatland CO2 and CH4 Fluxes With Water Table Modulating Temperature Sensitivity
Source: Glob Chang Biol. 2026 Sep 27;32(10):e71129. doi: 10.1111/gcb.71129 (PMC13617173; doi:10.1111/gcb.71129)
Supplement: Supplementary file 1 [file GCB-32-e71129-s001.docx]

**Supplementary Information**

**Water table and vegetation shape peatland CO₂ and CH₄ fluxes with water table modulating temperature sensitivity**

*Heyu Chen et al.*

**Contents**

**Fig. S1**	AIC comparison of candidate flux–WTL models

**Fig. S2**	Dependence of wet-end GHGE change on CH₄ weighting

**Fig. S3**	Flux and emission-side GHGE by graminoid presence

**Fig. S4**	Comparison of sliding-window and hierarchical estimates of apparent Q₁₀

**Fig. S5**	Site support for within-site temperature slopes across the water-table gradient

**Fig. S6**	Flux and apparent Q₁₀ across the water-table–temperature space, grouped by graminoid presence

**Fig. S7**	Water-table modulation of apparent CH₄ Q₁₀ under a three-state vegetation classification

**Fig. S8**	Effect of a GPP correction on the water-table response of emission-side GHGE

**Fig. S9**	Breakpoint structure and location under three weighting schemes

**Fig. S10**	Sliding-window apparent Q₁₀ profiles

**Fig. S11**	Funnel plot of site-level CH₄ sensitivity estimates

**Table S1**	Selected structural models for the flux–water-table relationships

**Table S2**	Comparison of binary and three-state vegetation classifications for the WTL interaction

**Table S3**	Wetland-type composition of the three vegetation states

**Table S4**	Robustness of the within-bog graminoid-presence contrast in CH₄ Q₁₀ modulation

**Table S5**	Supported water-table ranges under three informative-site thresholds

**Table S6**	Apparent Q₁₀ at the peat surface by gas and vegetation class

**Table S7**	Breakpoint structure under four treatments of extreme fluxes

**Table S8**	Eddy-covariance sites used for the GPP–WTL sensitivity analysis

**Table S9**	Site-by-site vegetation curation

**Supplementary Figures**

**Supplementary Fig. S1 | AIC comparison of candidate flux–WTL models.** Five response forms were evaluated for CH₄ and ecosystem respiration (R_eco_): linear, exponential, quadratic, one-breakpoint and two-breakpoint models. Models used signed-log-transformed fluxes, site as a random intercept and weights of ln(N) + 1. Bars show ΔAIC relative to the minimum AIC within each gas and vegetation class. a, CH₄, all peatlands; b, CH₄ in graminoid-dominated (upper) and non-graminoid-dominated (lower) peatlands; c, R_eco_ (labelled CO₂), all peatlands; d, R_eco_ in graminoid-dominated (upper) and non-graminoid-dominated (lower) peatlands. The retained model is labelled “Selected” in bold. For breakpoint selection, linear, one-breakpoint and two-breakpoint models were compared sequentially, and an additional breakpoint was retained only when it reduced AIC by >10. Because breakpoint selection favoured the simpler model unless AIC improved by >10, the retained model was not always the model with the minimum AIC. Exponential and quadratic forms are shown for comparison only and were not eligible for selection. In the R_eco_ graminoid-dominated cell (d, upper), both forms have lower AIC than the retained linear model, by 1.5 (exponential) and 6.0 (quadratic). The ΔAIC > 10 rule was used only for breakpoint selection. The dashed line marks ΔAIC = 10, and the shaded area lies beyond it. Bars longer than ΔAIC = 80 are truncated for display; the truncated bars in a are ΔAIC = 129.3 (linear) and 84.4 (exponential). The ΔAIC of the selected model is 0 in a and in b (graminoid), 2.9 in b (non-graminoid), 4.0 in c, 9.1 in d (graminoid) and 6.2 in d (non-graminoid).

**Supplementary Fig. S2 | Dependence of wet-end GHGE change on CH₄ weighting.** **a**, Fitted emission-side GHGE against water-table level for all peatlands, graminoid-dominated peatlands and non-graminoid-dominated peatlands, shown at four CH₄ weights in GHGE = R_eco_ + w × CH₄ (w = 28, 34, 84 and 120). **b**, Change in fitted GHGE (mg CO₂-eq m⁻² h⁻¹; an absolute rather than percentage change) between the peat surface and the wet limit of each drawn curve (+15.0 cm for all peatlands and graminoid-dominated peatlands, +10.4 cm for non-graminoid-dominated peatlands), shown as a continuous function of CH₄ weight. At w = 28, the value for all peatlands (−85.6 mg CO₂-eq m⁻² h⁻¹) corresponds to the 28.4% decline reported in the main text. Negative values indicate that GHGE declines towards the wet end, and positive values indicate that it rises. The horizontal line in b marks no change, and the grey dashed horizontal line in a marks zero GHGE. Open circles mark the CH₄ weight at which the wet-end change becomes zero, at w = 88 for all peatlands and w = 76 for non-graminoid-dominated peatlands. The weight axis spans w = 5 to 120. The graminoid curve does not reach zero anywhere in that range, so no circle is shown for it. Marked weights are 28 (the IPCC AR5 100-year value used throughout), 34 (the 100-year value including climate–carbon feedbacks) and 84 (the 20-year value). Curves use the same fitted models and wet-end truncation as Fig. 2c and Fig. 3c.

**Supplementary Fig. S3 | Flux and emission-side GHGE by graminoid presence.** Counterpart of Fig. 3, with observations grouped by graminoid presence rather than dominant vegetation class. **a**, CH₄ flux. **b,** Ecosystem respiration. **c,** Emission-side GHGE at w = 28. The same models, transformations, weighting and truncation rules as in Fig. 3 were used. The observations are those analysed in Fig. 3, re-partitioned by graminoid presence rather than replaced. Observations whose graminoid presence could not be resolved are excluded. This removes 180 CH₄ observations from eight sites and 137 R_eco_ and paired GHGE observations from seven sites. Five of these sites leave the comparison entirely (CH₄, 227 to 222 sites; R_eco_, 147 to 142 sites; GHGE, 111 to 106 sites). Graminoids were present in 6,485 CH₄ observations from 146 sites, 2,590 R_eco_ observations from 81 sites and 2,194 paired GHGE observations from 62 sites, and were absent or negligible in 5,052 CH₄ observations from 94 sites, 2,525 R_eco_ observations from 71 sites and 2,024 paired GHGE observations from 53 sites. A site name can contribute to both groups when different microforms, plots or sampling locations were classified separately, so the two site counts sum to more than the number of sites in each panel. Plotting conventions follow Fig. 3 and Fig. 2, except that orange denotes graminoids present and blue denotes graminoids absent or negligible. For the graminoid-absent CH₄ group, the second breakpoint (−2.5 cm) was selected within a search range that ends at 0 cm, the 95th percentile of this group's WTL. Its profile likelihood, evaluated up to the 98th percentile (+4.3 cm), is highest at the wettest profiled value (+4 cm). Its support set (+3 to +4 cm) therefore does not contain the fitted value, and no band is drawn. This breakpoint and the slope above it are not interpreted. Profile-likelihood support was also disjoint for the first CH₄ breakpoint of the graminoid-absent group, whose support set is a single profiled value at −21 cm together with −19 to −15 cm, so that only the second piece is wide enough to be drawn; for the R_eco_ breakpoint of the graminoid-present group (−50.2 to −45.2 cm and −32.2 to −17.2 cm; both pieces are shaded); and for the second R_eco_ breakpoint of the graminoid-absent group, for which the main support interval (−8.1 to −1.1 cm) is shown and a second piece at the wet edge of the profiled range (+1.9 to +3.9 cm) is not.

**Supplementary Fig. S4 | Comparison of sliding-window and hierarchical estimates of apparent Q₁₀.** Apparent Q₁₀ is shown on a logarithmic scale against water-table level for CH₄ and ecosystem respiration (R_eco_) in graminoid- and non-graminoid-dominated peatlands. Each panel is drawn only over that class's supported range in Fig. 4c, and the window estimates are computed at 1-cm steps within that range. Blue and red lines show estimates from 30-cm sliding windows using within-site temperature variation and absolute temperature, respectively. Black lines show hierarchical within-site models fitted separately to each gas and vegetation class, with linear (solid) or smooth (dashed) WTL modulation of the within-site temperature coefficient. Because each class is fitted with its own modulation, these curves are not the shared-modulation model shown in Fig. 4c, and their levels differ from those in Supplementary Table S6. Relative to the linear form, the smooth form changed AIC by −2.57 (CH₄, graminoid; edf = 3.90), −1.23 (CH₄, non-graminoid; edf = 3.11), +0.41 (R_eco_, graminoid; edf = 2.00) and −2.64 (R_eco_, non-graminoid; edf = 3.52). Shaded bands show 95% confidence intervals for the two model estimates, in grey for the linear fit and orange for the smooth fit. In the R_eco_ graminoid-dominated panel the smooth modulation reduces to the linear form (edf = 2.00), so its dashed line and orange band lie under the solid line and grey band. The sliding-window estimates carry no interval; they are shown for comparison only and were not used for formal inference.

**Supplementary Fig. S5 | Site support for within-site temperature slopes across the water-table gradient.** The number of sites with at least three observations and at least 2 °C of within-site temperature range, among the positive-flux observations used in the Q₁₀ models (sample sizes as in Fig. 4c), within a 30-cm window centred at each water-table position, shown separately for each gas and vegetation class. Support was evaluated here at 1-cm steps between −60 and +20 cm, so the vertical edges at these limits mark the evaluation range rather than a loss of support. The combined-subset support quoted in Fig. 5 was found on the full −80 to +30 cm domain and reaches −63 cm. The dotted line marks the minimum support threshold of 20 sites. Curves in Fig. 4c are shown only over the longest continuous WTL range meeting this threshold.

**Supplementary Fig. S6 | Flux and apparent Q₁₀ across the water-table–temperature space, grouped by graminoid presence.** This figure is the graminoid-presence counterpart of Fig. 4a–c. **a,** CH₄ flux. **b,** Ecosystem respiration (R_eco_) flux. Colour shows the mean signed-log-transformed flux in each bin. Panels a and b show the observations of Fig. 4a,b re-partitioned by graminoid presence, excluding those whose presence could not be resolved, over the same −65 to +25 cm window (CH₄: present n = 5,125 from 119 sites, absent or negligible n = 4,223 from 65 sites; R_eco_: present n = 1,903 from 70 sites, absent or negligible n = 2,050 from 52 sites). **c,** Apparent Q₁₀ against water-table level from the hierarchical model. Binning, colour scaling, the grey dashed visual guides at WTL = −40, −20 and 0 cm, the model structure, the 95% confidence ribbons, the faint 30-cm sliding-window within-site estimates and the ≥20-site display rule follow Fig. 4. The printed text in c gives the change in apparent Q₁₀ per 30 cm rise in WTL, with its 95% confidence interval and P value, and the numbers of positive-flux observations and sites used in the Q₁₀ model. On the presence axis, the structure with group-specific WTL modulation has a slightly lower AIC than the shared-modulation structure drawn here (by 0.3 for CH₄ and 2.3 for R_eco_); the shared structure is retained for comparison with Fig. 4. This whole-dataset comparison is not adjusted for wetland type. Among non-graminoid-dominant observations, the graminoid-present group is much more often bog than the graminoid-absent group. Bog-only comparisons of the WTL modulation of Q₁₀ are shown in Fig. 4d (non-graminoid-dominated bogs, by graminoid presence, for CH₄ and R_eco_) and Supplementary Fig. S7b (all bogs, three vegetation states, CH₄ only).

**Supplementary Fig. S7 | Water-table modulation of apparent CH₄ Q₁₀ under a three-state vegetation classification.** Three states were fitted together: graminoid-dominant, non-graminoid-dominated (NGRA) with graminoids present and NGRA with graminoids absent or negligible (labelled “NGRA, present” and “NGRA, absent” in the panels). **a,** All peatlands, shown on the absolute Q₁₀ scale. Wetland type is not controlled. **b,** Bogs only, shown on a relative scale and normalised to 1 at −10 cm, so wetland type is held constant by restriction. In this all-bog model, the WTL modulation of CH₄ Q₁₀ was 8.96 times larger (95% CI 3.43–23.40, P = 7.5 × 10⁻⁶) where graminoids were present than where they were absent. Within each panel, curves are drawn over one window common to all three states, running from the highest of the three states' 2nd water-table percentiles to the lowest of their 98th percentiles (a, −47.8 to +2.2 cm; b, −41.4 to +0.7 cm), so that the three curves are compared over the same interval. Shaded bands show 95% confidence intervals, which in b have zero width at −10 cm because the curves are normalised there. Sample sizes are given in each panel in the matching colours. The −10 cm reference in b is for display only and does not represent a hydrological threshold. In b, the upper confidence band of the graminoid-absent state is truncated at the axis limit between −41.4 and −31.7 cm.

**Supplementary Fig. S8 | Effect of a GPP correction on the water-table response of emission-side GHGE.** Emission-side and GPP-corrected GHGE against water-table level for **a,** all peatlands (n = 4,556 paired observations from 120 sites), b, graminoid-dominated peatlands (n = 1,517 from 44 sites) and c, non-graminoid-dominated peatlands (n = 2,838 from 76 sites). The emission-side curves are the w = 28 models of Fig. 2c and Fig. 3c, fitted to the same observations with the same weights and drawn from −80 cm to the 99th percentile of WTL in each panel (+15.0, +15.0 and +10.4 cm). The GPP correction subtracts the water-table-dependent component of GPP, β × WTL, in which β is a constant GPP–WTL slope of −1.52 mg CO₂ m⁻² h⁻¹ cm⁻¹. This slope is the mean of the eddy-covariance GPP–WTL slopes fitted separately for graminoid-dominated (−0.84, 33 towers) and non-graminoid-dominated (−1.88, 15 towers) sites of the screened tower compilation, weighted by the number of paired observations in each vegetation class (Methods Sections 2.1.6 and 2.2.6; Supplementary Table S8). The printed text in each panel gives the range (maximum minus minimum) of the corrected curve over the plotted WTL range as a proportion of the range of the emission-side curve. Both curves decline across the whole plotted range in every panel, so neither reaches an internal minimum. The grey dashed horizontal line in each panel marks zero GHGE. The slope is a single constant estimated from tower-level models. Leaving individual towers out of those models moves the retained fraction in a between 64% and 87%, so these percentages indicate the size of the correction rather than a precise value.

**Supplementary Fig. S9 | Breakpoint structure and location under three weighting schemes.** Selected breakpoint locations for CH₄ and ecosystem respiration (R_eco_), globally and by dominant vegetation class, under three weighting schemes: ln(N) + 1 (primary), unweighted and √N. All other model settings were unchanged, including the sequential linear → one-breakpoint → two-breakpoint comparison, the ΔAIC > 10 selection rule, site random intercept and WTL domain. The selected structure was the same under all three schemes in every cell, and the fitted breakpoints moved by at most 1.5 cm. Where the linear model was selected, the cell is labelled and no breakpoint is plotted. The grey dashed vertical line marks the peat surface (WTL = 0 cm). Symbols within each row are offset vertically for legibility only, and rows labelled CO₂ show R_eco_.

**Supplementary Fig. S10 | Sliding-window apparent Q₁₀ profiles.** Apparent Q₁₀ estimated in 30-cm windows along the water-table gradient from the slope of log flux on absolute soil temperature, shown for CH₄ and ecosystem respiration (R_eco_; panels labelled CO₂) in each dominant vegetation class. Windows were centred at 0.5-cm steps between −60 and +20 cm and retained only when they contained at least 300 observations, so profile extents differ from the supported ranges of Fig. 4c. Shaded bands show 95% confidence intervals. Horizontal dashed lines and printed values show a single WTL-independent apparent Q₁₀ for each flux (CH₄ 2.41; R_eco_ 3.27), estimated with the same absolute-temperature model fitted to all positive-flux observations of both vegetation classes across −80 to +30 cm. They are shown for orientation only and differ from the fixed Q₁₀ used in Fig. 5c. These profiles are retained for descriptive comparison. Neighbouring windows overlap substantially and the contributing sites change along the WTL gradient, so these profiles were not used for formal inference. This estimator blends within-site and between-site temperature responses. Supplementary Fig. S4 compares this estimator with a within-site window estimator and with hierarchical within-site fits for each gas and vegetation class. The windows there are also 30 cm wide, but they are evaluated at 1-cm steps and only over the supported ranges of Fig. 4c, so they cover part of the range shown here.

**Supplementary Fig. S11 | Funnel plot of site-level CH₄ sensitivity estimates.** Site-level CH₄ sensitivity to water-table level, estimated as the slope of signed-log CH₄ flux against WTL, plotted against precision (1/SE). Point size indicates the number of observations behind each estimate and colour indicates dominant vegetation class. The solid vertical line marks the inverse-variance weighted pooled mean. The inner darker region, bounded by the dashed lines, shows the 95% pseudo-confidence limits and the outer lighter region shows the 99% limits. Slopes were fitted to the CH₄ observations of Fig. 3a, separately for each dominant vegetation class at a site and only where that class had at least 10 observations and a water-table standard deviation above 1 cm (171 estimates from 163 of the 227 sites). Estimates whose precision exceeded the upper quartile by more than three interquartile ranges (10) or whose slope lay more than three interquartile ranges beyond the quartiles (26) were excluded. Both rules act on quantities that the funnel is used to inspect: the precision rule removes the ten most precise estimates and the slope rule trims the tails. The remaining 135 estimates from 130 sites are shown. The pooled mean drawn here is that of the plotted estimates (0.0009 slog units per cm); over all 171 eligible estimates it is 0.0002.

**Supplementary Tables**

**Supplementary Table S1 | Selected structural models for the flux–water-table relationships.** Linear and piecewise-linear mixed models were fitted to signed-log-transformed fluxes over the −80 to +30 cm WTL domain, using weights of ln(N) + 1 and site as a random intercept. An additional breakpoint was retained only when it reduced AIC by >10 relative to the next simpler model. Breakpoint intervals are profile-likelihood support intervals; slope intervals are 95% confidence intervals. Marginal R² represents fixed effects only, whereas conditional R² includes fixed and random effects. τ = breakpoint (cm); β = segment slope (signed-log units per cm), numbered from the driest segment. ᵃ The profile support set is disjoint; the interval shown is the component containing the estimate.

| **Response and vegetation class** | **n** | **Sites** | **Selected structure** | **τ₁ (cm)** | **τ₁ support** | **τ₂ (cm)** | **τ₂ support** | **β₁** | **β₂** | **β₃** | **Marginal R²** | **Conditional R²** |
| --- | --- | --- | --- | --- | --- | --- | --- | --- | --- | --- | --- | --- |
| CH₄, all peatlands | 12,305 | 242 | Two breakpoints | −25.4 | −31.7 to −22.7 | −8.4 | −10.4 to −6.4 | 0.001 [−0.001, 0.003] | 0.024 [0.021, 0.026] | 0.007 [0.004, 0.010] | 0.036 | 0.422 |
| CH₄, graminoid-dominated | 4,283 | 113 | Two breakpoints | −31.3 | −34.4 to −29.4 | −9.8 | −11.4 to −7.4 ᵃ | −0.005 [−0.009, −0.001] | 0.025 [0.021, 0.029] | 0.005 [0.001, 0.010] | 0.037 | 0.481 |
| CH₄, non-graminoid-dominated | 7,434 | 132 | One breakpoint | −27.1 | −38.6 to −21.6 | — | — | 0.002 [0.000, 0.004] | 0.014 [0.012, 0.016] | — | 0.030 | 0.356 |
| R_eco_, all peatlands | 5,530 | 158 | One breakpoint | −26.5 | −29.0 to −23.0 | — | — | −0.012 [−0.015, −0.009] | −0.030 [−0.033, −0.027] | — | 0.081 | 0.529 |
| R_eco_, graminoid-dominated | 1,662 | 49 | Linear | — | — | — | — | −0.021 [−0.024, −0.017] | — | — | 0.078 | 0.335 |
| R_eco_, non-graminoid-dominated | 3,590 | 108 | One breakpoint | −25.0 | −28.0 to −21.0 | — | — | −0.011 [−0.014, −0.008] | −0.034 [−0.038, −0.030] | — | 0.081 | 0.608 |

**Supplementary Table S2 | Comparison of binary and three-state vegetation classifications for the WTL interaction.** The three-state classification is nested within both binary classifications: the dominance axis merges the two non-graminoid-dominant states, whereas the presence axis merges the graminoid-dominant and graminoid-present states. Each row compares the three-state interaction with each binary interaction using likelihood-ratio tests of nested models that differ by one parameter and are fitted to the same observations. Observations whose graminoid presence could not be resolved are excluded, so n is smaller than in Fig. 3 and Table 1. In the flux rows the interaction is a single linear WTL slope for each state, not the piecewise structure of Supplementary Table S1; in the Q₁₀ rows it is the within-site temperature × WTL coefficient for each state. The comparison is made on the interaction with water table, not on the level: main effects remain at three-state resolution so that only the WTL interaction differs between models. ΔAIC is reported for reference and is the AIC of the binary model minus the AIC of the three-state model, so positive values favour three states and the single negative value favours the binary classification; the ΔAIC > 10 rule used for breakpoint selection is not applied here. For R_eco_ Q₁₀ modulation the three-state classification improved on both binary classifications (P = 9.0 × 10⁻⁴ and P = 0.018), although no WTL modulation of R_eco_ Q₁₀ was detected in the shared model (Table 1).

| **Quantity** | **n** | **Sites** | **ΔAIC vs dominance** | **χ² vs dominance** | **P vs dominance** | **ΔAIC vs presence** | **χ² vs presence** | **P vs presence** |
| --- | --- | --- | --- | --- | --- | --- | --- | --- |
| CH₄ flux vs water table | 11,537 | 222 | 46.91 | 48.91 | 2.7 × 10⁻¹² | 55.56 | 57.56 | 3.3 × 10⁻¹⁴ |
| R_eco_ flux vs water table | 5,115 | 142 | 5.61 | 7.61 | 0.0058 | 11.04 | 13.04 | 3.0 × 10⁻⁴ |
| CH₄ Q₁₀ modulation vs water table | 7,822 | 168 | 1.61 | 3.61 | 0.0575 | −0.62 | 1.38 | 0.24 |
| R_eco_ Q₁₀ modulation vs water table | 4,126 | 114 | 9.01 | 11.01 | 9.0 × 10⁻⁴ | 3.60 | 5.60 | 0.0179 |
| CH₄ Q₁₀ modulation, bogs only | 2,875 | 58 | 13.85 | 15.85 | 6.8 × 10⁻⁵ | 1.97 | 3.97 | 0.0464 |

**Supplementary Table S3 | Wetland-type composition of the three vegetation states.** Numbers of observations and sites in each vegetation state, the numbers of observations in bogs, in fens and in all other classes, and the bog and fen proportions. Counts are for the 13,121 observations within the −80 to +30 cm WTL domain whose dominance and graminoid presence were resolved, so they are smaller than the full-database counts in Fig. 1c. Observations were counted as bog or fen when the recorded wetland classification named a bog or a fen, and were counted as other otherwise. The other class therefore holds classes such as peat swamp, tundra, marsh and forest swamp, together with 200 observations for which no wetland classification was recorded. A site name can contribute to more than one vegetation state when different microforms, plots or sampling locations were classified separately, so the per-state site counts sum to 279 for 256 unique sites. The non-graminoid-dominant state with graminoids present is strongly concentrated in bogs, whereas the graminoid-absent state contains a much smaller bog fraction. Graminoid-dominant observations are predominantly fens, so the dominance axis is also partly confounded with wetland type. Vegetation state and wetland type are therefore partly confounded at the whole-dataset scale. Comparisons that separate these effects are shown within bogs in Supplementary Fig. S7b and Supplementary Table S4.

| **Vegetation state** | **n** | **Sites** | **Bog** | **Fen** | **Other** | **% bog** | **% fen** |
| --- | --- | --- | --- | --- | --- | --- | --- |
| Graminoid-dominant | 4,455 | 118 | 699 | 2,729 | 1,027 | 15.7 | 61.3 |
| Non-graminoid, graminoids present | 2,885 | 52 | 2,484 | 36 | 365 | 86.1 | 1.2 |
| Non-graminoid, graminoids absent or negligible | 5,781 | 109 | 1,393 | 1,059 | 3,329 | 24.1 | 18.3 |

**Supplementary Table S4 | Robustness of the within-bog graminoid-presence contrast in CH₄ Q₁₀ modulation.** The reported ratio compares the WTL modulation of apparent CH₄ Q₁₀ over a 30 cm rise between graminoid-present and graminoid-absent sites within non-graminoid-dominated bogs; a ratio of 1 indicates no contrast. The main Fig. 4d analysis uses a basis dimension of k = 8 for the group-specific smooth WTL term, whereas robustness fits use k = 6 to ensure convergence of all leave-one-out models. This changes the corresponding site-level baseline from ×4.65 to ×4.95, so the two blocks are reported separately and are not directly comparable. Eleven of the 24 peatland complexes contain more than one site, so leaving one site out cannot test complex-level clustering whereas leaving one complex out can. Two site names contribute to both presence groups because their microforms or plots were classified separately, so the 48 unique sites of this subset yield 50 arm-specific entries. The Sites column reports those 50 arm entries in the k = 8 block, matching the CH₄ site counts of Fig. 4d (22 present and 28 absent or negligible), and unique site names in the k = 6 block, 48 in the baseline fit and fewer where a restriction removes sites.

| **Check** | **n** | **Sites** | **Ratio (95% CI)** | **P** |
| --- | --- | --- | --- | --- |
| Main analysis (k = 8) |  |  |  |  |
| Site random effect, as in Fig. 4d | 2,341 | 50 | 4.65 (1.86–11.61) | 0.001 |
| Random effect keyed on plot (site × presence arm) | 2,341 | 50 | 4.58 (1.85–11.35) | 0.001 |
| Robustness fits (k = 6) |  |  |  |  |
| Baseline, all bogs | 2,341 | 48 | 4.95 (2.05–11.98) | 3.9 × 10⁻⁴ |
| Random effect at the peatland-complex level | 2,341 | 48 | 3.87 (1.60–9.32) | 0.0026 |
| Nested peatland-complex and site random effects | 2,341 | 48 | 4.57 (1.83–11.39) | 0.0011 |
| Drop the single site holding 35% of the absent arm | 2,104 | 47 | 7.63 (2.86–20.34) | 4.8 × 10⁻⁵ |
| Plain “bog” subtype only | 2,183 | 38 | 6.13 (2.22–16.93) | 4.7 × 10⁻⁴ |
| Water table restricted to −35 to 0 cm | 2,124 | 45 | 5.43 (1.52–19.47) | 0.0094 |
| Water table restricted to −30 to 0 cm | 2,040 | 44 | 3.91 (0.98–15.66) | 0.054 |
| Water table restricted to −40 to +5 cm | 2,244 | 47 | 4.94 (1.62–15.09) | 0.0051 |
| Leave one site out, 48 fits | — | — | 2.17 to 7.63; no interval includes 1 | ≤ 0.036 |
| Leave one peatland complex out, 24 fits | — | — | 2.17 to 10.97; no interval includes 1 | ≤ 0.036 |

**Supplementary Table S5 | Supported water-table ranges under three informative-site thresholds.** An informative site had at least three observations and at least 2 °C of within-site temperature variation within a 30-cm window centred at each WTL position. The supported range is the longest continuous WTL interval, in cm relative to the peat surface, meeting the specified minimum number of informative sites. Support was evaluated between −60 and +20 cm; limits at −60 or +20 cm are set by this evaluation range. The primary analysis uses a threshold of 20 sites; thresholds of 12 and 30 are shown as sensitivity bounds.

| **Gas** | **Vegetation class** | **≥ 12 sites** | **≥ 20 sites (used)** | **≥ 30 sites** |
| --- | --- | --- | --- | --- |
| CH₄ | Graminoid | −56 to +20 | −39 to +17 | −30 to +16 |
| CH₄ | Non-graminoid | −60 to +16 | −55 to +15 | −45 to +12 |
| R_eco_ | Graminoid | −37 to +15 | −25 to +12 | −10 to −2 |
| R_eco_ | Non-graminoid | −60 to +12 | −55 to +8 | −45 to +4 |

**Supplementary Table S6 | Apparent Q₁₀ at the peat surface by gas and vegetation class.** Class-specific apparent Q₁₀ at WTL = 0 cm from the hierarchical model used for Table 1. Vegetation classes have separate Q₁₀ levels, whereas the WTL modulation is shared across classes. Absolute level differences were sensitive to the random-effect grouping; the R_eco_ level contrast of ×1.47 under the published site names fell to ×0.97 when sites were grouped by coordinates and wetland class, and levels are therefore not interpreted ecologically. *n* denotes positive-flux observations used in the Q₁₀ models. Supported ranges are class-specific (at least 20 informative sites, evaluated at 1-cm steps between −60 and +20 cm, as in Fig. 4c and Supplementary Table S5); the ranges of −63 to +20 cm for CH₄ and −63 to +15 cm for R_eco_ given for Table 1 refer to the combined vegetation subset evaluated between −80 and +30 cm.

| **Gas** | **Vegetation class** | **Q₁₀ at WTL = 0 cm (95% CI)** | **n** | **Sites** | **Supported range (cm)** |
| --- | --- | --- | --- | --- | --- |
| CH₄ | Graminoid | 3.74 (2.80–5.00) | 2,921 | 90 | −39 to +17 |
| CH₄ | Non-graminoid | 2.85 (2.17–3.73) | 5,056 | 96 | −55 to +15 |
| R_eco_ | Graminoid | 3.90 (3.25–4.67) | 1,180 | 41 | −25 to +12 |
| R_eco_ | Non-graminoid | 2.65 (2.27–3.09) | 3,081 | 86 | −55 to +8 |

**Supplementary Table S7 | Breakpoint structure under four treatments of extreme fluxes.** The primary analysis retains all eligible observations. Sensitivity analyses apply 1.5 × interquartile range (IQR) removal, 3 × IQR removal or winsorisation at the 1.5 × IQR bounds, each applied to the signed-log flux of the observations in the analysis cell. All other model settings follow the primary flux–WTL analysis. The selected breakpoint structure is unchanged across the four treatments, although breakpoint locations shift by up to 5.6 cm, in every case remaining within the profile-likelihood support interval of the primary estimate. Because removal and winsorisation can act unevenly along the WTL gradient, the treated datasets are used only as sensitivity analyses. τ = breakpoint location.

| **Analysis** | **Treatment** | **n** | **Sites** | **Selected structure** | **τ₁ (cm)** | **τ₂ (cm)** |
| --- | --- | --- | --- | --- | --- | --- |
| CH₄, all peatlands | None (primary) | 12,305 | 242 | Two breakpoints | −25.4 | −8.4 |
| R_eco_, all peatlands | None (primary) | 5,530 | 158 | One breakpoint | −26.5 | — |
| CH₄, graminoid-dominated | None (primary) | 4,283 | 113 | Two breakpoints | −31.3 | −9.8 |
| CH₄, non-graminoid-dominated | None (primary) | 7,434 | 132 | One breakpoint | −27.1 | — |
| R_eco_, graminoid-dominated | None (primary) | 1,662 | 49 | Linear | — | — |
| R_eco_, non-graminoid-dominated | None (primary) | 3,590 | 108 | One breakpoint | −25.0 | — |
| CH₄, all peatlands | Removal at 1.5 × IQR | 12,033 | 242 | Two breakpoints | −31.0 | −7.0 |
| R_eco_, all peatlands | Removal at 1.5 × IQR | 5,384 | 155 | One breakpoint | −26.5 | — |
| CH₄, graminoid-dominated | Removal at 1.5 × IQR | 4,258 | 113 | Two breakpoints | −31.3 | −10.3 |
| CH₄, non-graminoid-dominated | Removal at 1.5 × IQR | 7,067 | 132 | One breakpoint | −26.2 | — |
| R_eco_, graminoid-dominated | Removal at 1.5 × IQR | 1,630 | 49 | Linear | — | — |
| R_eco_, non-graminoid-dominated | Removal at 1.5 × IQR | 3,494 | 105 | One breakpoint | −25.0 | — |
| CH₄, all peatlands | Removal at 3 × IQR | 12,302 | 242 | Two breakpoints | −25.4 | −8.4 |
| R_eco_, all peatlands | Removal at 3 × IQR | 5,516 | 158 | One breakpoint | −26.5 | — |
| CH₄, graminoid-dominated | Removal at 3 × IQR | 4,283 | 113 | Two breakpoints | −31.3 | −9.8 |
| CH₄, non-graminoid-dominated | Removal at 3 × IQR | 7,381 | 132 | One breakpoint | −26.2 | — |
| R_eco_, graminoid-dominated | Removal at 3 × IQR | 1,659 | 49 | Linear | — | — |
| R_eco_, non-graminoid-dominated | Removal at 3 × IQR | 3,586 | 108 | One breakpoint | −24.5 | — |
| CH₄, all peatlands | Winsorised | 12,305 | 242 | Two breakpoints | −26.9 | −7.9 |
| R_eco_, all peatlands | Winsorised | 5,530 | 158 | One breakpoint | −26.5 | — |
| CH₄, graminoid-dominated | Winsorised | 4,283 | 113 | Two breakpoints | −31.3 | −9.8 |
| CH₄, non-graminoid-dominated | Winsorised | 7,434 | 132 | One breakpoint | −27.1 | — |
| R_eco_, graminoid-dominated | Winsorised | 1,662 | 49 | Linear | — | — |
| R_eco_, non-graminoid-dominated | Winsorised | 3,590 | 108 | One breakpoint | −25.0 | — |

**Supplementary Table S8 | Eddy-covariance sites used for the GPP–WTL sensitivity analysis.** The table lists 74 wetland flux-tower sites: 72 compiled from AmeriFlux, the FLUXNET-CH₄ Community Product, UK-SCAPE and the Netherlands peatland network, and two from individual publications. Sixty-one sites were retained after quality screening. Vegetation was classified using the same dominance-based framework as for the chamber dataset. The GPP–WTL slope used in Supplementary Fig. S8 was fitted to the 48 of these 61 sites with concurrent GPP, water-table and soil-temperature records inside the fitting window (33 graminoid-dominated and 15 non-graminoid-dominated). Source gives the network or publication from which each site was compiled; full references for the two publications are given below the table. Status gives whether a site was used in the GPP–WTL slope model (48 sites), retained after screening but without concurrent soil-temperature or water-table records inside the fitting window (13 sites) or excluded during screening (13 sites). IGBP class is the International Geosphere–Biosphere Programme land-cover class: WET, permanent wetland; CRO, cropland; EBF, evergreen broadleaf forest; ENF, evergreen needleleaf forest; GRA, grassland. In the Vegetation class column, GRA denotes graminoid-dominated and NGRA denotes non-graminoid-dominated.

| **Site ID** | **Site name** | **Country** | **Latitude** | **Longitude** | **Elevation (m)** | **IGBP class** | **Source** | **Vegetation class** | **Status** |
| --- | --- | --- | --- | --- | --- | --- | --- | --- | --- |
| BR-Npw | Northern Pantanal Wetland | Brazil | −16.5 | −56.41 | 120 | WET | AmeriFlux | NGRA | Slope model |
| CA-ARB | Attawapiskat River Bog | Canada | 52.695 | −83.945 | 90 | WET | AmeriFlux | NGRA | Slope model |
| CA-ARF | Attawapiskat River Fen | Canada | 52.7 | −83.955 | 90 | WET | AmeriFlux | GRA | Slope model |
| CA-BOU | Bouleau Peatland | Canada | 50.524 | −63.206 | 108 | WET | AmeriFlux | NGRA | Slope model |
| CA-DB2 | Delta Burns Bog 2 | Canada | 49.119 | −122.995 | 4 | WET | AmeriFlux | NGRA | Slope model |
| CA-DBB | Delta Burns Bog 1 | Canada | 49.129 | −122.985 | 4 | WET | AmeriFlux | NGRA | Slope model |
| CA-DSM | Delta Salt Marsh | Canada | 49.089 | −122.895 | 2.41 | WET | AmeriFlux | GRA | Slope model |
| CA-SCB | Scotty Creek Bog | Canada | 61.31 | −121.3 | 280 | WET | FLUXNET-CH4 | NGRA | Slope model |
| CN-HY | Hongyuan Peatland | China | 32.767 | 102.5 | 3510 | WET | Peng et al. (2021) | GRA | Slope model |
| CN-RGQ | Riganqiao Fen | China | 33.1043 | 102.6515 | 3460 | WET | Liu et al. (2019) | GRA | Slope model |
| DE-Hte | Hütelmoor | Germany | 54.21 | 12.18 | 0 | WET | FLUXNET-CH4 | GRA | Slope model |
| DE-Zrk | Zarnekow | Germany | 53.88 | 12.89 | 0 | WET | FLUXNET-CH4 | GRA | Slope model |
| FI-Lom | Lompolojänkkä | Finland | 68 | 24.2 | 269 | WET | FLUXNET-CH4 | GRA | Slope model |
| FI-Si2 | Siikaneva 2 (bog) | Finland | 61.84 | 24.2 | 162 | WET | FLUXNET-CH4 | GRA | Slope model |
| FI-Sii | Siikaneva 1 (fen) | Finland | 61.83 | 24.19 | 162 | WET | FLUXNET-CH4 | NGRA | Slope model |
| ID-Pag | Palangkaraya | Indonesia | −2.345 | 114.036 | 20 | EBF | FLUXNET-CH4 | NGRA | Slope model |
| JP-BBY | Bibai | Japan | 43.32 | 141.75 | 7 | WET | FLUXNET-CH4 | GRA | Slope model |
| JP-Mse | Mase Rice Paddy | Japan | 36.054 | 140.027 | 15 | CRO | FLUXNET-CH4 | GRA | Slope model |
| KR-CRK | Cheorwon Rice Paddy | South Korea | 38.201 | 127.25 | 100 | CRO | FLUXNET-CH4 | GRA | Slope model |
| MY-MLM | Maludam | Malaysia | 1.454 | 111.149 | 6 | EBF | FLUXNET-CH4 | NGRA | Slope model |
| NZ-Kop | Kopuatai | New Zealand | −37.39 | 175.55 | 3 | WET | FLUXNET-CH4 | GRA | Slope model |
| SE-Deg | Degerö Stormyr | Sweden | 64.18 | 19.56 | 270 | WET | FLUXNET-CH4 | GRA | Slope model |
| US-ALQ | Allequash Creek | USA | 46.03 | −89.61 | 500 | WET | AmeriFlux | GRA | Excluded |
| US-CRT | Curtice Walter-Berger Cropland | USA | 41.63 | −83.35 | 180 | CRO | AmeriFlux | — | Excluded |
| US-Cms | Central Marsh Barrow | USA | 71.32 | −156.61 | 5 | WET | AmeriFlux | GRA | Excluded |
| US-EDN | Eden Landing | USA | 37.62 | −122.11 | 1.65 | WET | AmeriFlux | GRA | Excluded |
| US-Elm | Everglades Shark River Slough | USA | 25.55 | −80.78 | 0.77 | WET | AmeriFlux | GRA | Retained |
| US-Esm | Everglades Taylor Slough | USA | 25.44 | −80.59 | 0 | WET | AmeriFlux | GRA | Excluded |
| US-EvM | Everglades Saltwater Intrusion | USA | 25.35 | −80.38 | 0.33 | WET | AmeriFlux | GRA | Excluded |
| US-HB1 | North Inlet Saltmarsh | USA | 33.49 | −79.19 | 0 | WET | AmeriFlux | GRA | Excluded |
| US-Ho1 | Howland Forest | USA | 45.2 | −68.74 | 60 | ENF | AmeriFlux | NGRA | Excluded |
| US-Hsm | Hill Slough Marsh | USA | 38.24 | −122.02 | 0 | WET | AmeriFlux | NGRA | Slope model |
| US-Los | Lost Creek | USA | 46.08 | −89.98 | 480 | WET | AmeriFlux | NGRA | Slope model |
| US-MAC | MacArthur Agro-Ecology | USA | 27.164 | −81.187 | 15 | WET | FLUXNET-CH4 | GRA | Retained |
| US-Myb | Mayberry Wetland | USA | 38.05 | −121.77 | −4 | WET | AmeriFlux | GRA | Retained |
| US-NC4 | NC Alligator River | USA | 35.79 | −75.9 | 1 | WET | AmeriFlux | NGRA | Slope model |
| US-ONA | Florida Pine Flatwoods | USA | 27.39 | −81.95 | 27.1 | WET | AmeriFlux | NGRA | Excluded |
| US-OWC | Old Woman Creek | USA | 41.38 | −82.51 | 174 | WET | AmeriFlux | GRA | Retained |
| US-PAS | Florida Paspalum Pasture | USA | 27.39 | −81.95 | 27.1 | GRA | AmeriFlux | GRA | Excluded |
| US-Sne | Sherman Island Restored Wetland | USA | 38.04 | −121.75 | −5 | WET | AmeriFlux | NGRA | Slope model |
| US-Srr | Suisun Marsh Rush Ranch | USA | 38.2 | −122.03 | 0 | WET | AmeriFlux | GRA | Excluded |
| US-StJ | St Jones Reserve | USA | 39.09 | −75.44 | 0 | WET | AmeriFlux | GRA | Slope model |
| US-Tw1 | Twitchell West Pond | USA | 38.107 | −121.647 | −5 | WET | AmeriFlux | GRA | Slope model |
| US-Tw4 | Twitchell East End | USA | 38.103 | −121.641 | −5 | WET | AmeriFlux | GRA | Retained |
| US-Tw5 | East Pond Wetland | USA | 38.11 | −121.64 | −5 | WET | AmeriFlux | GRA | Retained |
| US-Twt | Twitchell Island Rice | USA | 38.109 | −121.653 | −5 | CRO | AmeriFlux | GRA | Slope model |
| US-Uaf | Univ Alaska Fairbanks | USA | 64.87 | −147.86 | 155 | WET | FLUXNET-CH4 | NGRA | Slope model |
| US-WPT | Winous Point North Marsh | USA | 41.47 | −83 | 174 | WET | FLUXNET-CH4 | GRA | Slope model |
| NL_ank_pt | Ankeveen Paludiculture | Netherlands | 52.25 | 5.1 | −1.3 | WET | Netherlands network | GRA | Slope model |
| NL_zeg_pt | Zegveld Paludiculture | Netherlands | 52.14 | 4.84 | −2.4 | WET | Netherlands network | GRA | Retained |
| NL_cam | Camphuys Semi-natural | Netherlands | 53.15 | 6.58 | 0.6 | WET | Netherlands network | GRA | Retained |
| NL_ilp_pt | Ilperveld Semi-natural | Netherlands | 52.44 | 4.95 | −0.5 | WET | Netherlands network | GRA | Slope model |
| NL_onl | Onlanden Semi-natural | Netherlands | 53.18 | 6.52 | −1.8 | WET | Netherlands network | GRA | Retained |
| NL_wrw_sr | Weerribben Semi-natural Reed | Netherlands | 52.77 | 5.93 | −0.6 | WET | Netherlands network | GRA | Slope model |
| NL_wrw_ow | Weerribben/Duinigermeer Lake | Netherlands | 52.72 | 6 | −0.5 | WET | Netherlands network | GRA | Retained |
| NL_dem_nt | Demmerik Wet Grassland | Netherlands | 52.2 | 4.95 | −2 | WET | Netherlands network | GRA | Slope model |
| NL_asd_mp | Assendelft Pasture WIS | Netherlands | 52.48 | 4.74 | −1.8 | WET | Netherlands network | GRA | Slope model |
| NL_law_ms | Langeweide Pasture WIS | Netherlands | 52.04 | 4.79 | −2 | WET | Netherlands network | GRA | Slope model |
| NL_hoc | Hommerts C Pasture | Netherlands | 52.99 | 5.68 | −0.9 | WET | Netherlands network | GRA | Slope model |
| NL_hoh | Hommerts H Pasture | Netherlands | 52.99 | 5.67 | −1 | WET | Netherlands network | GRA | Slope model |
| NL_ldc | Lytse Deelen C Pasture | Netherlands | 53.05 | 5.94 | −0.9 | WET | Netherlands network | GRA | Slope model |
| NL_ldh | Lytse Deelen H Pasture | Netherlands | 53.05 | 5.94 | −1 | WET | Netherlands network | GRA | Slope model |
| NL_buo | De Burd C Pasture | Netherlands | 53.1 | 5.87 | −1.2 | WET | Netherlands network | GRA | Slope model |
| NL_buw | De Burd T Pasture | Netherlands | 53.1 | 5.86 | −0.9 | WET | Netherlands network | GRA | Slope model |
| UK_AF_HN | Anglesey 2 Short Fen | United Kingdom | 53.3103 | −4.2911 | 62.59 | WET | UK-SCAPE | GRA | Slope model |
| UK_AF_LN | Anglesey 1 Tall Fen | United Kingdom | 53.3097 | −4.3004 | 62.59 | WET | UK-SCAPE | GRA | Retained |
| UK_BD_OG | Sheepdrove Organic | United Kingdom | 51.5302 | −1.4819 | 180.86 | CRO | UK-SCAPE | GRA | Excluded |
| UK_EF_GF | Great Fen Grassland | United Kingdom | 52.4705 | −0.193 | −0.24 | WET | UK-SCAPE | GRA | Slope model |
| UK_EF_NE | Woodwalton Grassland | United Kingdom | 52.46 | −0.1851 | 2.54 | WET | UK-SCAPE | GRA | Slope model |
| UK_EF_SA | Redmere 1 Cropland | United Kingdom | 52.443 | 0.4195 | −1.02 | CRO | UK-SCAPE | GRA | Retained |
| UK_EF_SH | Redmere 2 Cropland | United Kingdom | 52.445 | 0.4237 | −1.02 | CRO | UK-SCAPE | GRA | Retained |
| UK_LA_IG | Myerscough Grassland | United Kingdom | 53.8546 | −2.7796 | 10.95 | GRA | UK-SCAPE | GRA | Excluded |
| UK_NP_BB | Moor House Blanket Bog | United Kingdom | 54.695 | −2.3882 | 564.73 | WET | UK-SCAPE | NGRA | Slope model |
| UK_SL_EG | Tadham Moor Grassland | United Kingdom | 51.2073 | −2.8285 | 3.54 | WET | UK-SCAPE | GRA | Slope model |

References for the Source column:

Liu, X., Zhu, D., Zhan, W., Chen, H., Zhu, Q., Hao, Y., Liu, W., & He, Y. (2019). Five-year measurements of net ecosystem CO₂ exchange at a fen in the Zoige peatlands on the Qinghai-Tibetan Plateau. *Journal of Geophysical Research: Atmospheres*, *124*(22), 11803–11818. https://doi.org/10.1029/2019JD031429

Peng, H., Chi, J., Yao, H., Guo, Q., Hong, B., Ding, H., Zhu, Y., Wang, J., & Hong, Y. (2021). Methane emissions offset net carbon dioxide uptake from an alpine peatland on the eastern Qinghai-Tibetan Plateau. *Journal of Geophysical Research: Atmospheres*, *126*(19), e2021JD034671. https://doi.org/10.1029/2021JD034671

**Supplementary Table S9 | Site-by-site vegetation curation.** Vegetation dominance and graminoid presence assigned to each of the 285 sites in the database; n is the number of observations contributed by that row. Twenty-one site names appear in more than one row, because a name can cover more than one microform, plot or sampling location, giving 316 rows in total. Curation status distinguishes labels retained from the source database (209 rows) from those re-read from the source publication by hand (72 rows) and those re-read from the source PDF by automated extraction with adversarial verification (35 rows). Source vegetation descriptions, species lists, the assigned labels, and the curation status, confidence, source and reason are released as columns of the dataset. Across the full database, 4,558 observations were classified as graminoid-dominated and 9,310 as non-graminoid-dominated; 775 were uncertain, mixed or lacked an assignable dominant type. Within the −80 to +30 cm analysis domain, 734 observations from 21 sites remained in these unresolved categories and were excluded from vegetation comparisons. Unknown in the Graminoid column denotes graminoid presence that could not be resolved. In the Dominance column, GRA denotes graminoid-dominated, NGRA denotes non-graminoid-dominated, Mixed denotes mixed vegetation without a dominant group, Uncertain denotes dominance that could not be resolved from the source, and None denotes vegetation-free plots that were not assigned a dominance class.

| **Site** | **Country** | **Latitude** | **Longitude** | **Wetland type** | **n** | **Dominance** | **Graminoid** | **Curation** |
| --- | --- | --- | --- | --- | --- | --- | --- | --- |
| APEX Beta Active Margin AMCH site | USA | 64.6962 | −148.3201 | Fen | 29 | Mixed | Present | re-read from the source publication |
| APEX Beta Permafrost Plateau PPC site | USA | 64.6963 | −148.3219 | Permafrost Bog | 50 | NGRA | Present | re-read from the source publication |
| APEX Beta Permafrost Plateau PPCH site | USA | 64.6963 | −148.3219 | Permafrost Bog | 50 | NGRA | Present | re-read from the source publication |
| Abisko Stordalen Mire | Sweden | 68.367 | 19.05 | peatland complex | 4 | GRA | Present | retained from the source database |
| Abisko Stordalen Mire | Sweden | 68.367 | 19.05 | peatland complex | 2 | NGRA | Absent-negligible | retained from the source database |
| Ahvensalo Hollows Site | Finland | 62.8333 | 30.8833 | Bog | 5 | NGRA | Present | re-read from the source publication |
| Ahvensalo Hummocks Site | Finland | 62.8333 | 30.8833 | Bog | 5 | NGRA | Present | re-read from the source publication |
| Ahvensalo Sphagnum angustifolium Site | Finland | 62.8333 | 30.8833 | Bog | 5 | NGRA | Present | re-read from the source publication |
| Ahvensalo Sphagnum fuscum Site | Finland | 62.8333 | 30.8833 | Bog | 5 | NGRA | Present | re-read from the source publication |
| Alan Batu peat swamp forest | Malaysia | 1.45 | 111.14 | peat swamp | 18 | NGRA | Absent-negligible | retained from the source database |
| Arboretum muskeg, Alaska | USA | 64.867 | −147.85 | tundra | 18 | NGRA | Absent-negligible | retained from the source database |
| Arboretum muskeg, Alaska | USA | 64.867 | −147.85 | tundra | 9 | GRA | Present | retained from the source database |
| Auchencorth Moss south of Edinburgh, Scotland | UK | 55.785 | −3.246 | bog | 35 | GRA | Present | retained from the source database |
| Barrow BEN site | USA | 71.29 | −156.6034 | Wet Tundra | 1 | GRA | Present | retained from the source database |
| Barrow BEO site | USA | 71.31 | −156.5965 | Wet Tundra; Permafrost Bog; Dry Tundra | 15 | GRA | Present | retained from the source database |
| Barrow BEO site | USA | 71.2811 | −156.6122 | Permafrost Bog | 3 | NGRA | Absent-negligible | retained from the source database |
| Barrow BES site | USA | 71.02 | −156.6123 | Wet Tundra | 4 | GRA | Present | retained from the source database |
| Barrow Dry site | USA | 71.28 | −156.6167 | Dry Tundra | 1 | GRA | Present | retained from the source database |
| Barrow Environmental Observatory, near Utqiagvik (Barrow), Alaska | USA | 71.323 | −156.615 | tundra | 592 | NGRA | Absent-negligible | retained from the source database |
| Barrow Environmental Observatory, near Utqiagvik (Barrow), Alaska | USA | 71.323 | −156.615 | tundra | 99 | GRA | Present | retained from the source database |
| Barrow Flat Center | USA | 71.2811 | −156.613 | Permafrost Bog | 1 | GRA | Present | retained from the source database |
| Barrow Flooded site | USA | 71.28 | −156.6167 | Wet Tundra | 2 | GRA | Present | retained from the source database |
| Barrow Low Center | USA | 71.2812 | −156.6116 | Wet Tundra | 1 | GRA | Present | retained from the source database |
| Barrow Wet site | USA | 71.28 | −156.6167 | Wet Tundra | 1 | GRA | Present | retained from the source database |
| Barrow moist site | USA | 71.28 | −156.6167 | Moist Tundra | 1 | GRA | Present | retained from the source database |
| Barrow thaw lake — Intermediate microsite | USA | 71.32 | −156.62 | Arctic tundra / drained thaw-lake basin | 27 | Uncertain | Present | re-read from the source PDF |
| Barrow thaw lake — Polygon rims | USA | 71.32 | −156.62 | Arctic tundra / drained thaw-lake basin | 27 | NGRA | Present | re-read from the source PDF |
| Barrow thaw lake — Wet sedge microsite | USA | 71.32 | −156.62 | Arctic tundra / drained thaw-lake basin | 27 | GRA | Present | retained from the source database |
| Bernadouze peatland | France | 42.802 | 1.4236 | Forest Swamp | 31 | GRA | Present | retained from the source database |
| Beverly Swamp | Canada | 43.38 | −80.25 | peat swamp | 179 | NGRA | Absent-negligible | retained from the source database |
| Bibai wetland – Sasa invasion site (Sa) | Japan | 43.317 | 141.8 | Ombrotrophic peatland | 13 | NGRA | Absent-negligible | retained from the source database |
| Bibai wetland – Sphagnum-dominated site (Sp) | Japan | 43.317 | 141.8 | Ombrotrophic peatland | 13 | NGRA | Present | re-read from the source PDF |
| Bouleau peatland Hollow site | Canada | 50.5167 | −63.2 | Bog | 10 | NGRA | Absent-negligible | re-read from the source publication |
| Bouleau peatland Hummock Lichen site | Canada | 50.5167 | −63.2 | Bog | 10 | NGRA | Absent-negligible | re-read from the source publication |
| Bouleau peatland Hummock Sphagnum site | Canada | 50.5167 | −63.2 | Bog | 10 | NGRA | Absent-negligible | re-read from the source publication |
| Bouleau peatland Lawn site | Canada | 50.5167 | −63.2 | Bog | 10 | NGRA | Present | re-read from the source publication |
| CC – Hummock | Ecuador | −0.3191 | −78.1997 | — | 1 | NGRA | Unknown | re-read from the source PDF |
| CC – Lawn | Ecuador | −0.4947 | −78.276 | — | 1 | Mixed | Present | re-read from the source PDF |
| CN_Sanjiang_Carex_lasiocarpa_zone | China | 47.58 | 133.62 | marsh | 17 | GRA | Present | retained from the source database |
| CN_Sanjiang_Carex_pseudo-curaica_zone | China | 47.58 | 133.62 | marsh | 17 | GRA | Present | retained from the source database |
| CN_Sanjiang_Deyeuxia_zone | China | 47.58 | 133.62 | marsh | 17 | GRA | Present | retained from the source database |
| Carex lasiocarpa–dominated herbaceous peatland (Sanjiang Plain) | China | 47.5 | 133.35 | marsh | 61 | GRA | Present | retained from the source database |
| Carex muliensis hollow stand | China | 33.934 | 102.867 | fen | 9 | GRA | Present | retained from the source database |
| Central Kalimantan | Indonesia | −1.867 | 113.7 | peat swamp | 332 | NGRA | Absent-negligible | retained from the source database |
| Central Kalimantan | Indonesia | −1.867 | 113.7 | peat swamp | 107 | GRA | Present | retained from the source database |
| Central Kalimantan – K-FOR (secondary peat forest) | Indonesia | −2.828 | 111.813 | Peat swamp | 18 | NGRA | Absent-negligible | retained from the source database |
| Central Kalimantan – K-OP-(oil palm) | Indonesia | −2.828 | 111.813 | Peat swamp | 18 | NGRA | Absent-negligible | retained from the source database |
| Central alpine Carex pamirensis marsh control | China | 37.58 | 101.33 | Marsh | 7 | GRA | Present | retained from the source database |
| Central alpine Carex pamirensis marsh low WT | China | 37.58 | 101.33 | Marsh | 7 | GRA | Present | retained from the source database |
| Chersky – Waterlogged wetland (CW) | Russia | 68.53 | 161.43 | floodplain | 36 | GRA | Present | retained from the source database |
| China Zoige Plateau – Riganqiao fen (alpine sedge fen) | China | 33.1 | 102.65 | fen | 23 | GRA | Present | retained from the source database |
| Controls peat bog site | UK | 57.46 | −3.62 | bog | 270 | NGRA | Present | re-read from the source PDF |
| Cultivated peat soil (bare soil experimental field) | Finland | 60.79 | 23.54 | fen | 19 | None | Absent-negligible | re-read from the source publication |
| Cwm-y-Glo forested swamp | UK | 52.9772 | −3.7822 | Acidic bog | 12 | NGRA | Absent-negligible | retained from the source database |
| D16 (Control – drained) | USA | 35.751 | −76.5102 | bog | 11 | NGRA | Absent-negligible | retained from the source database |
| Degero Stormyr_oligotrophic minerogenic mire complex | Sweden | 64.1833 | 19.55 | Bog | 13 | NGRA | Present | re-read from the source publication |
| Denmark Eastern Denmark – Permanent Grassland (E-PG) | Denmark | 56.337 | 10.377 | fen | 19 | GRA | Present | re-read from the source PDF |
| Denmark Northern Jutland – Permanent Grassland (N-PG) | Denmark | 56.881 | 10.204 | fen | 19 | Mixed | Present | re-read from the source PDF |
| Denmark Western Jutland – Permanent Grassland (W-PG) | Denmark | 55.943 | 8.5 | fen | 19 | GRA | Present | re-read from the source PDF |
| Drained Cottongrass pine bog | Finland | 61.7833 | 24.3167 | bog | 20 | NGRA | Present | re-read from the source PDF |
| Drained Tall sedge fen (T1 drained) | Finland | 61.7833 | 24.3167 | fen | 9 | GRA | Present | retained from the source database |
| Drained pine site | Sweden | 57.13 | 14.75 | bog | 27 | NGRA | Present | re-read from the source PDF |
| Drained pocosin | USA | 35.9 | −76.25 | bog | 42 | NGRA | Absent-negligible | retained from the source database |
| Drained site 1 | China | 33.917 | 102.817 | Marsh | 9 | GRA | Present | retained from the source database |
| Drained site 2 | China | 33.917 | 102.817 | Marsh | 9 | GRA | Present | retained from the source database |
| Drained site 3 | China | 33.917 | 102.817 | Marsh | 9 | GRA | Present | retained from the source database |
| Drained spruce site with old trees | Sweden | 57.13 | 14.75 | bog | 30 | NGRA | Absent-negligible | retained from the source database |
| Drained spruce site with young trees | Sweden | 57.13 | 14.75 | bog | 31 | NGRA | Absent-negligible | retained from the source database |
| Drayton Valley peat extraction area | Canada | 53.557 | −114.736 | — | 158 | NGRA | Absent-negligible | retained from the source database |
| East Anglia BS with high SOC | UK | 52.0833 | 1 | fen | 11 | NGRA | Absent-negligible | retained from the source database |
| East Anglia – SOC HIGH farm (70% SOM) | UK | 52.0833 | 1 | fen | 11 | NGRA | Absent-negligible | retained from the source database |
| East Anglia – SOC LOW farm (20% SOM) | UK | 52.0833 | 1 | fen | 13 | NGRA | Absent-negligible | retained from the source database |
| East Anglia – SOC MED farm (35% SOM) | UK | 52.0833 | 1 | fen | 22 | NGRA | Absent-negligible | retained from the source database |
| East Flanders Moss – n-pris (near-pristine bog) | UK | 56.1333 | −4.3 | bog | 37 | NGRA | Unknown | re-read from the source publication |
| Eleocharis valleculosa hollow stand | China | 33.934 | 102.867 | fen | 9 | GRA | Present | retained from the source database |
| Ess-soo peatland | Estonia | 58.874 | 26.254 | bog | 227 | NGRA | Present | re-read from the source publication |
| Ess-soo peatland | Estonia | 57.914 | 26.697 | bog | 222 | GRA | Present | re-read from the source publication |
| Ess-soo peatland | Estonia | 58.427 | 24.786 | bog | 93 | NGRA | Absent-negligible | re-read from the source publication |
| Estonia Tässi peatland – Bare Peat (BP) | Estonia | 58.55 | 25.87 | Bog | 20 | NGRA | Absent-negligible | retained from the source database |
| Estonia Tässi peatland – ResH (High WTL Restoration) | Estonia | 58.55 | 25.87 | Bog | 20 | NGRA | Absent-negligible | re-read from the source PDF |
| Estonia Tässi peatland – ResL (Low WTL Restoration) | Estonia | 58.55 | 25.87 | Bog | 20 | NGRA | Absent-negligible | re-read from the source PDF |
| Finland Ilomantsi – Drained fen grassland (hay meadow) | Finland | 62.667 | 30.8333 | fen | 20 | GRA | Present | re-read from the source PDF |
| Flanders Moss – DP (Drained + Planted) | UK | 56.1333 | −4.3 | bog | 24 | NGRA | Unknown | re-read from the source publication |
| Flanders Moss – uDP (Undrained + Planted) | UK | 56.1333 | −4.3 | bog | 34 | NGRA | Unknown | re-read from the source publication |
| Flanders Moss – uDuP (Undrained + Unplanted) | UK | 56.1333 | −4.3 | bog | 37 | Uncertain | Unknown | re-read from the source publication |
| Fort Simpson region, NWT, Canada | Canada | 61.8 | −121.4 | — | 8 | GRA | Present | retained from the source database |
| Fort Simpson region, NWT, Canada | Canada | 61.8 | −121.4 | — | 6 | NGRA | Absent-negligible | retained from the source database |
| Fort Simpson region, NWT, Canada | Canada | 61.8 | −121.4 | — | 4 | NGRA | Present | re-read from the source PDF |
| Great Hing’an Mountains – Continuous permafrost peatland – Eriophorum-dominated plots (EP) | China | 52.49 | 122.86 | bog | 36 | GRA | Present | retained from the source database |
| Great Hing’an Mountains – Continuous permafrost peatland – Shrub–moss dominated plots (SP) | China | 52.49 | 122.86 | bog | 36 | NGRA | Absent-negligible | retained from the source database |
| HDPF Dense Forest Area | Sweden | 64.183 | 19.575 | peat swamp | 78 | NGRA | Absent-negligible | retained from the source database |
| HDPF Sparse Forest Area | Sweden | 64.183 | 19.575 | peat swamp | 78 | NGRA | Present | re-read from the source PDF |
| Halssiaapa wetland | Finland | 67.367 | 26.653 | Fen | 693 | GRA | Present | retained from the source database |
| Heaven Swamp | Canada | 56.955 | −111.555 | peat swamp | 117 | NGRA | Absent-negligible | retained from the source database |
| Heavily degraded peat swamp | Peru | −3.8333 | −73.3142 | peat swamp | 72 | NGRA | Absent-negligible | retained from the source database |
| Hongyuan Zoige peatland – drainage experiment | China | 32.81 | 102.56 | Degraded alpine peatland / Marsh meadow | 45 | GRA | Present | retained from the source database |
| Huahu Lake margin site, Ruoergai Plateau | China | 33.9167 | 102.867 | Natural alpine seasonal-flooded peat marsh | 21 | GRA | Present | retained from the source database |
| Hydrologically restored | USA | 35.751 | −76.5102 | bog | 11 | NGRA | Absent-negligible | retained from the source database |
| Intact peat swamp forest | Peru | −3.8333 | −73.3142 | peat swamp | 72 | NGRA | Absent-negligible | retained from the source database |
| Iskoras thaw slump site | Norway | 69.5667 | 25.05 | Wet Tundra | 12 | NGRA | Present | re-read from the source publication |
| Jabiren tropical peatland – fallow peat under natural vegetation | Indonesia | −2.5 | 114.17 | bog | 3 | NGRA | Absent-negligible | retained from the source database |
| Jamb Lowland paddy field | Indonesia | −1.242 | 103.616 | paddy | 12 | GRA | Present | retained from the source database |
| Jambi Cassava field | Indonesia | −1.24 | 103.622 | peat swamp | 12 | NGRA | Absent-negligible | retained from the source database |
| Jambi Drained forest | Indonesia | −1.242 | 103.616 | peat swamp | 12 | NGRA | Unknown | re-read from the source publication |
| Jambi Upland paddy field | Indonesia | −1.241 | 103.66 | paddy | 12 | GRA | Present | retained from the source database |
| Kaamanen Flark | Finland | 69.08 | 27.17 | Fen | 4 | GRA | Present | re-read from the source publication |
| Kaamanen Lawn | Finland | 69.08 | 27.17 | Fen | 4 | NGRA | Present | re-read from the source publication |
| Kaamanen String | Finland | 69.08 | 27.17 | Bog | 4 | NGRA | Absent-negligible | re-read from the source publication |
| Katingan shrub site | Indonesia | −2.74 | 113.15 | peat swamp | 9 | NGRA | Absent-negligible | retained from the source database |
| Kevo, Skalluvaara Open peatland | Finland | 69.8167 | 27.1667 | Fen | 18 | GRA | Present | retained from the source database |
| Kevo, Skalluvaara Palsa | Finland | 69.8167 | 27.1667 | Permafrost Bog | 8 | GRA | Present | retained from the source database |
| La Grande Rivière | Canada | 48.3968 | −64.5021 | rich fen | 13 | NGRA | Absent-negligible | retained from the source database |
| La Grande Rivière | Canada | 48.3968 | −64.5021 | rich fen | 3 | NGRA | Present | re-read from the source PDF |
| La Grande Rivière | Canada | 48.3968 | −64.5021 | rich fen | 2 | GRA | Present | retained from the source database |
| Lakkasuo Drained site | Finland | 61.792 | 24.2761 | Bog | 14 | NGRA | Present | re-read from the source publication |
| Lakkasuo Natural site | Finland | 61.792 | 24.2761 | Bog | 14 | NGRA | Present | re-read from the source publication |
| Lakkasuo oligo PS (shrubs and Carex sp. removed) | Finland | 61.78 | 24.3 | fen | 186 | NGRA | Absent-negligible | retained from the source database |
| Lakkasuo oligo PSCD control | Finland | 61.78 | 24.3 | fen | 590 | GRA | Present | retained from the source database |
| Lakkasuo oligo PSC (peat + Sphagnum + sedges, shrubs removed) | Finland | 61.78 | 24.3 | fen | 279 | GRA | Present | retained from the source database |
| Lakkasuo oligo bare peat | Finland | 61.78 | 24.3 | fen | 216 | NGRA | Absent-negligible | retained from the source database |
| Lakkasuo peatland complex Wet subsite | Finland | 61.783 | 24.3 | Bog | 20 | GRA | Present | retained from the source database |
| Lakkasuo peatland complex in Orivesi | Finland | 61.783 | 24.3 | bog | 14 | NGRA | Absent-negligible | retained from the source database |
| Lakkasuo peatland complex in Orivesi | Finland | 61.783 | 24.3 | bog | 6 | GRA | Present | retained from the source database |
| Lakkasuo peatland complex in Orivesi | Finland | 61.783 | 24.3 | bog | 4 | NGRA | Unknown | re-read from the source PDF |
| Lower Kolyma River floodplain | Russia | 68.5 | 161.4 | floodplain wetlands | 3 | GRA | Present | retained from the source database |
| Luanhaizi Wetland – Tibetan Plateau | China | 37.583 | 101.333 | Alpine permafrost peatland / Marsh | 7 | GRA | Present | retained from the source database |
| Lutose CA-LUT | Canada | 59.4845 | −117.1765 | Permafrost Bog | 7 | NGRA | Absent-negligible | re-read from the source publication |
| Lutose CA-LUT | Canada | 59.4845 | −117.1765 | Bog | 6 | Mixed | Present | re-read from the source publication |
| Lutose CA-LUT | Canada | 59.4845 | −117.1765 | Bog | 5 | NGRA | Present | re-read from the source publication |
| Lutose extreme-rich fen | Canada | 59.4832 | −117.1811 | Fen | 4 | GRA | Present | retained from the source database |
| Lutose intermediate bog | Canada | 59.4845 | −117.1765 | Bog | 24 | Mixed | Present | re-read from the source publication |
| Lutose mature bog | Canada | 59.4845 | −117.1765 | Bog | 26 | NGRA | Present | re-read from the source publication |
| Lutose poor fen | Canada | 59.4844 | −117.175 | Fen | 4 | GRA | Present | retained from the source database |
| Lutose rich fen | Canada | 59.4842 | −117.1747 | Fen | 4 | GRA | Present | retained from the source database |
| Lutose young bog | Canada | 59.4845 | −117.1765 | Bog | 28 | Mixed | Present | re-read from the source publication |
| Maludam National Park – Alan Batu Forest (ABt) | Malaysia | 1.45 | 111.133 | Peat Swamp | 33 | NGRA | Absent-negligible | retained from the source database |
| Maludam National Park – Alan Bunga Forest (ABg) | Malaysia | 1.45 | 111.15 | Peat Swamp | 33 | NGRA | Absent-negligible | retained from the source database |
| Maludam National Park – Mixed Peat Swamp Forest (MPS) | Malaysia | 1.417 | 111.1167 | Peat Swamp | 33 | NGRA | Absent-negligible | retained from the source database |
| Mannikjarve-mixopeat | Estonia | 58.8749 | 26.251 | Bog | 6 | NGRA | Present | re-read from the source publication |
| Mer Bleue Bog – Chamaedaphne-dominated community site | Canada | 45.41 | −75.52 | ombrotrophic bog | 24 | NGRA | Absent-negligible | retained from the source database |
| Mer Bleue Bog – Eriophorum-dominated community site | Canada | 45.41 | −75.52 | ombrotrophic bog | 24 | GRA | Present | retained from the source database |
| Mer Bleue Bog – Maianthemum & Ledum dominated community site | Canada | 45.41 | −75.52 | ombrotrophic bog | 24 | NGRA | Absent-negligible | retained from the source database |
| Mer Bleue Peatland, Ottawa | Canada | 45.41 | −75.48 | ombrotrophic bog | 50 | GRA | Present | retained from the source database |
| Mer Bleue Peatland, Ottawa | Canada | 45.41 | −75.48 | ombrotrophic bog | 50 | NGRA | Absent-negligible | retained from the source database |
| Middle Taiga Zone large hollow site | Russia | 60.9 | 68.7 | Bog | 8 | GRA | Present | retained from the source database |
| Middle Taiga Zone small ridge site | Russia | 60.9 | 68.7 | Bog | 8 | GRA | Present | retained from the source database |
| Migneint acidic bog | UK | 52.9772 | −3.7822 | Acidic bog | 12 | NGRA | Absent-negligible | retained from the source database |
| Mixed peat swamp forest (Forest, F) | Malaysia | 2.817 | 111.85 | Peat Swamp | 12 | NGRA | Absent-negligible | retained from the source database |
| Mixed-type peat swamp forest (Sebangau, Central Kalimantan) | Indonesia | −2.33 | 113.9167 | peat swamp | 19 | NGRA | Absent-negligible | retained from the source database |
| Moderately degraded peat swamp | Peru | −3.8333 | −73.3142 | peat swamp | 72 | NGRA | Absent-negligible | retained from the source database |
| Mokré Louky – Gap | Czech Republic | 49.0166 | 14.77 | fen | 19 | GRA | Present | retained from the source database |
| Mokré Louky – Hummock | Czech Republic | 49.0166 | 14.77 | fen | 19 | GRA | Present | retained from the source database |
| Mukhrino Ira Hol site | Russia | 60.8918 | 68.6771 | Bog | 15 | NGRA | Absent-negligible | retained from the source database |
| Mukhrino Ira Ridge site | Russia | 60.8916 | 68.6769 | Bog | 4 | NGRA | Absent-negligible | retained from the source database |
| Mukhrino Ira WetHol site | Russia | 60.8919 | 68.6825 | Bog | 8 | NGRA | Absent-negligible | retained from the source database |
| Mukhrino bog E.hollow site | Russia | 60.8864 | 68.6919 | Bog | 4 | NGRA | Present | re-read from the source publication |
| Mukhrino bog Open bog site | Russia | 60.8999 | 68.6874 | Bog | 6 | NGRA | Present | re-read from the source publication |
| Mukhrino bog Ridge site | Russia | 60.8865 | 68.688 | Bog | 4 | NGRA | Absent-negligible | re-read from the source publication |
| Mukhrino bog Ryam site | Russia | 60.8951 | 68.6847 | Bog | 4 | NGRA | Absent-negligible | re-read from the source publication |
| Mukhrino bog S.hollow site | Russia | 60.8866 | 68.6879 | Bog | 4 | NGRA | Present | re-read from the source publication |
| Mukhrino bog Tall ryam site | Russia | 60.895 | 68.6965 | Bog | 6 | NGRA | Absent-negligible | re-read from the source publication |
| Naarasneva cutaway peatland | Finland | 62.8639 | 24.0838 | Fen | 459 | NGRA | Absent-negligible | retained from the source database |
| Natural pocosin (RF T2) | USA | 35.9 | −76.25 | bog | 14 | NGRA | Absent-negligible | retained from the source database |
| Natural raised bog | Denmark | 57.23 | 9.84 | bog | 32 | NGRA | Absent-negligible | retained from the source database |
| North Selangor | Malaysia | 3.55 | 101.067 | peat swamp | 243 | NGRA | Absent-negligible | retained from the source database |
| Northumberland 20 year | UK | 55.1667 | −2.05 | Peat swamp | 13 | NGRA | Absent-negligible | retained from the source database |
| Northumberland 30 year | UK | 55.1667 | −2.05 | Peat swamp | 17 | NGRA | Absent-negligible | retained from the source database |
| Northumberland OCFP | UK | 55.1667 | −2.05 | Peat swamp | 16 | NGRA | Absent-negligible | retained from the source database |
| Northumberland UG | UK | 55.1667 | −2.05 | Peat swamp | 15 | NGRA | Absent-negligible | retained from the source database |
| Nørre Å fen peatland – RCG experimental plots | Denmark | 55.73 | 10.1 | fen | 44 | GRA | Present | retained from the source database |
| Nørre Å fen peatland – RCG field experiment | Denmark | 56.44 | 9.68 | fen | 24 | GRA | Present | retained from the source database |
| Nørre Å fen peatland – Spring barley plots | Denmark | 55.73 | 10.1 | fen | 44 | NGRA | Absent-negligible | retained from the source database |
| Nørre Å river valley | Denmark | 55.73 | 10.1 | fen | 50 | GRA | Present | retained from the source database |
| Nørreå river valley of Central Jutland | Denmark | 56.48 | 9.68 | fen | 51 | GRA | Present | retained from the source database |
| Odderbæk – Restored Block 2 | Denmark | 55.926 | 9.926 | marsh | 27 | GRA | Present | retained from the source database |
| Oil palm plantation (1-year old) site | Indonesia | −2.79 | 111.81 | Peat swamp | 26 | NGRA | Absent-negligible | retained from the source database |
| Oil palm plantation (6-year old) site | Indonesia | −2.801 | 111.577 | Peat swamp | 26 | NGRA | Absent-negligible | retained from the source database |
| Oil palm plantation on drained peat | Malaysia | 2.817 | 111.93 | Peat Swamp | 12 | NGRA | Absent-negligible | retained from the source database |
| Oil palm plantation on peat far | Indonesia | −2.78 | 111.77 | peat swamp | 18 | NGRA | Absent-negligible | retained from the source database |
| Oil palm plantation on peat near | Indonesia | −2.78 | 111.77 | peat swamp | 18 | NGRA | Absent-negligible | retained from the source database |
| Orient Ombrotrophic raised bog | USA | 45.85 | −67.89 | Bog | 569 | NGRA | Present | re-read from the source PDF |
| Orient Ombrotrophic raised bog | USA | 45.85 | −67.89 | Bog | 160 | GRA | Present | retained from the source database |
| Ötztal range in Tyrol, Austria | Austria | 46.8333 | 11.05 | fen | 44 | GRA | Present | retained from the source database |
| Panama – San San Pond Sak 1 Palm Swamp | Panama | 8.967 | −82.117 | Peat Swamp | 6 | NGRA | Absent-negligible | retained from the source database |
| Panama – San San Pond Sak Mixed Forest | Panama | 9.167 | −82.25 | Peat Swamp | 7 | NGRA | Absent-negligible | retained from the source database |
| Peat swamp forest (Pesalat) site | Indonesia | −2.816 | 111.5333 | Peat swamp | 26 | NGRA | Absent-negligible | retained from the source database |
| Permanently Flooded Hollow | China | 33.917 | 102.817 | Marsh | 9 | GRA | Present | retained from the source database |
| Permanently Flooded Hummock | China | 33.917 | 102.817 | Marsh | 9 | GRA | Present | retained from the source database |
| Pointe-aux-Chenes WMA Brackish Marsh | USA | 29.5 | −90.44 | Brackish marsh | 5 | GRA | Present | retained from the source database |
| Prudhoe Bay, Alaska | USA | 70.229 | −148.419 | tundra | 60 | Uncertain | Present | re-read from the source PDF |
| Prudhoe Bay, Alaska | USA | 70.229 | −148.419 | tundra | 55 | GRA | Present | retained from the source database |
| Prudhoe Bay, Alaska | USA | 70.229 | −148.419 | tundra | 21 | Uncertain | Unknown | re-read from the source publication |
| Reed canary grass monoculture (RCG) | Denmark | 56.44 | 9.68 | Marsh | 120 | GRA | Present | retained from the source database |
| Restored Wetland (RW) | USA | 35.9061 | −76.1569 | Peat swamp | 11 | NGRA | Absent-negligible | retained from the source database |
| Restored pocosin (Rewetted shrub bog) B7 | USA | 35.9 | −76.25 | bog | 14 | NGRA | Absent-negligible | retained from the source database |
| Restored pocosin (Rewetted shrub bog) c2t2 | USA | 35.9 | −76.25 | bog | 14 | NGRA | Absent-negligible | retained from the source database |
| Restored pocosin (Rewetted shrub bog) d11 | USA | 35.9 | −76.25 | bog | 14 | NGRA | Absent-negligible | retained from the source database |
| Rewetted PSF (Katingan Mentaya Project) | Indonesia | −2.92 | 113.15 | Peat Swamp | 6 | NGRA | Absent-negligible | retained from the source database |
| Ränskälänkorpi Non_harvest | Finland | 61.18 | 25.267 | Forest Swamp | 97 | NGRA | Absent-negligible | retained from the source database |
| Ränskälänkorpi clearcut | Finland | 61.18 | 25.267 | Forest Swamp | 114 | NGRA | Absent-negligible | retained from the source database |
| Ränskälänkorpi select harvest | Finland | 61.18 | 25.267 | Forest Swamp | 112 | NGRA | Absent-negligible | retained from the source database |
| Sago palm plantation (Sago, S) | Malaysia | 2.783 | 111.83 | Peat Swamp | 12 | NGRA | Absent-negligible | retained from the source database |
| Sallie’s Fen, New Hampshire (temperate poor fen) | USA | 43.2083 | −71.0583 | fen | 19 | NGRA | Present | re-read from the source PDF |
| Salmisuo Mire, Eastern Finland Lawns site | Finland | 62.78 | 30.9333 | Fen | 15 | GRA | Present | retained from the source database |
| Samoylov Island site | Russia | 72.37 | 126.47 | Tundra | 21 | GRA | Present | retained from the source database |
| Sanjiang Plain saucer-shaped marsh – central belt Carex pseudocuraica | China | 47.58 | 133.625 | Marsh | 17 | GRA | Present | retained from the source database |
| Sanjiang Plain saucer-shaped marsh – intermediate belt Carex lasiocarpa | China | 47.58 | 133.625 | Marsh | 17 | GRA | Present | retained from the source database |
| Sarawak oil palm plantation – First rotation – drainage & collector ditches | Malaysia | 1.39 | 111.4 | Tropical peat swamp forest | 57 | NGRA | Absent-negligible | retained from the source database |
| Sarawak oil palm plantation – Second rotation – drainage & collector ditches | Malaysia | 1.41 | 111.432 | Tropical peat swamp forest | 29 | NGRA | Absent-negligible | retained from the source database |
| Sarawak – Mixed peat swamp forest | Malaysia | 2.817 | 111.85 | peat swamp | 12 | NGRA | Absent-negligible | retained from the source database |
| Sarawak – Sago plantation | Malaysia | 2.817 | 111.85 | peat swamp | 12 | NGRA | Absent-negligible | retained from the source database |
| Saskatchewan Bog | Canada | 55.85 | −107.6833 | bog | 7 | NGRA | Absent-negligible | retained from the source database |
| Saskatchewan Frost mound | Canada | 55.85 | −107.6833 | bog | 7 | NGRA | Absent-negligible | retained from the source database |
| Saskatchewan Internal lawn | Canada | 55.85 | −107.6833 | bog | 7 | NGRA | Present | re-read from the source PDF |
| Seasonally Flooded Hollow | China | 33.917 | 102.817 | Marsh | 9 | GRA | Present | retained from the source database |
| Seasonally Flooded Hummock | China | 33.917 | 102.817 | Marsh | 9 | GRA | Present | retained from the source database |
| Seasonally flooded hummock (S-hummock) | China | 33.92 | 102.82 | Marsh | 12 | GRA | Present | retained from the source database |
| Secondary peat swamp forest | Indonesia | −2.74 | 113.15 | peat swamp | 9 | NGRA | Absent-negligible | retained from the source database |
| Seida Bare Peat site | Russia | 67.057 | 62.9447 | Permafrost Bog | 7 | None | Absent-negligible | re-read from the source publication |
| Seida Carex fen site | Russia | 67.0582 | 62.946 | Fen | 7 | GRA | Present | re-read from the source publication |
| Seida Eriophorum fen site | Russia | 67.0583 | 62.9463 | Fen | 7 | GRA | Present | re-read from the source publication |
| Seida Tundra bog dry site | Russia | 67.0554 | 62.943 | Permafrost Bog | 7 | NGRA | Absent-negligible | re-read from the source publication |
| Seida Tundra bog moist site | Russia | 67.0555 | 62.9432 | Permafrost Bog | 7 | NGRA | Absent-negligible | re-read from the source publication |
| Seida Willow site | Russia | 67.0587 | 62.9443 | Fen | 7 | NGRA | Present | re-read from the source publication |
| Siikaneva C site | Finland | 61.8372 | 24.1967 | Bog | 48 | Uncertain | Unknown | re-read from the source publication |
| Siikaneva HHU site | Finland | 61.837 | 24.169 | Bog | 136 | NGRA | Absent-negligible | re-read from the source publication |
| Siikaneva HL site | Finland | 61.837 | 24.169 | Bog | 137 | NGRA | Present | re-read from the source publication |
| Siikaneva HO site | Finland | 61.837 | 24.169 | Bog | 132 | NGRA | Present | re-read from the source publication |
| Siikaneva HU site | Finland | 61.837 | 24.169 | Bog | 141 | NGRA | Absent-negligible | re-read from the source publication |
| Siikaneva L site | Finland | 61.837 | 24.169 | Bog | 136 | NGRA | Present | re-read from the source publication |
| Siikaneva MB site | Finland | 61.837 | 24.169 | Bog | 129 | Uncertain | Present | re-read from the source publication |
| Siikaneva bog | Finland | 61.86 | 24.33 | bog | 243 | NGRA | Present | re-read from the source publication |
| Siikaneva mixopeat site | Finland | 61.8449 | 24.2882 | Fen | 3 | NGRA | Present | re-read from the source publication |
| Siikaneva_P | Finland | 61.8333 | 24.2 | bog | 233 | None | Absent-negligible | re-read from the source publication |
| Siikaneva_PS | Finland | 61.8333 | 24.2 | bog | 380 | NGRA | Absent-negligible | re-read from the source publication |
| Siikaneva_PSV | Finland | 61.8333 | 24.2 | bog | 424 | NGRA | Present | re-read from the source publication |
| South Selangor | Malaysia | 2.867 | 101.433 | peat swamp | 246 | NGRA | Absent-negligible | retained from the source database |
| Stordalen A site | Sweden | 68.37 | 19.05 | Wet Tundra | 2 | GRA | Present | retained from the source database |
| Stordalen Abisko mixopeat_site | Sweden | 68.3453 | 19.0663 | Wet Tundra | 4 | NGRA | Present | re-read from the source publication |
| Stordalen Avni transect site | Sweden | 68.354 | 19.0505 | Permafrost Bog | 84 | GRA | Present | re-read from the source publication |
| Stordalen Avni transect site | Sweden | 68.354 | 19.0505 | Permafrost Bog | 51 | NGRA | Unknown | re-read from the source publication |
| Stordalen Avni transect site | Sweden | 68.354 | 19.0505 | Permafrost Bog | 13 | NGRA | Absent-negligible | re-read from the source publication |
| Stordalen B site | Sweden | 68.37 | 19.05 | Moist Tundra | 2 | GRA | Present | retained from the source database |
| Stordalen Mire | Sweden | 68.4 | 19.05 | Subarctic thawing mire | 8 | NGRA | Present | re-read from the source PDF |
| Stordalen Mire | Sweden | 68.35 | 19.167 | Subarctic thawing mire | 5 | GRA | Present | retained from the source database |
| Stordalen Mire | Sweden | 68.4 | 19.05 | Subarctic thawing mire | 2 | NGRA | Absent-negligible | retained from the source database |
| Stordalen Mire – Site A (wet minerotrophic) | Sweden | 68.35 | 19.03 | Minerotrophic wet fen | 4 | GRA | Present | retained from the source database |
| Stordalen Mire – Site B (ombrotrophic peat bog) | Sweden | 68.35 | 19.03 | Minerotrophic wet fen | 4 | GRA | Present | retained from the source database |
| Sweden – Asa Experimental Forest – Drained Alder Site | Sweden | 57.1333 | 14.75 | Peat swamp | 33 | NGRA | Absent-negligible | retained from the source database |
| Sweden – Asa Experimental Forest – Undrained Alder Site | Sweden | 57.1333 | 14.75 | Peat swamp | 36 | NGRA | Present | re-read from the source PDF |
| Sweden – Asa Forest – Drained Birch Site | Sweden | 57.1333 | 14.75 | Peat swamp | 45 | NGRA | Present | re-read from the source PDF |
| TQ peatland – Eriophorum dominated (EP) | China | 52.94 | 122.86 | bog | 32 | GRA | Present | retained from the source database |
| TQ peatland – Shrub–moss dominated (SP) | China | 52.94 | 122.86 | bog | 32 | NGRA | Absent-negligible | retained from the source database |
| Tambillos | Peru | −9.6894 | −77.2384 | — | 43 | NGRA | Present | re-read from the source PDF |
| Teslin collapse wetland | Canada | 60.083 | −132.367 | Fen | 74 | GRA | Present | retained from the source database |
| Tiksi Observatory in Sakha | Russia | 71.5943 | 128.8878 | wet fen; dry fen | 191 | GRA | Present | retained from the source database |
| Tiksi Observatory in Sakha | Russia | 71.5943 | 128.8878 | bog | 32 | NGRA | Absent-negligible | retained from the source database |
| Tobo, 40 km N of Uppsala, Sweden | Sweden | 60.25 | 17.617 | Minerotrophic mire | 76 | NGRA | Present | re-read from the source PDF |
| Transitional meadow–fen ecotone | Austria | 46.8333 | 11.05 | fen | 22 | GRA | Present | retained from the source database |
| Tundra Fen Abeille | Canada | 54.12 | −72.5017 | Fen | 6 | GRA | Present | retained from the source database |
| Turraun BarePeat | Ireland | 53.275 | −7.75 | fen | 62 | NGRA | Absent-negligible | retained from the source database |
| Turraun EC | Ireland | 53.275 | −7.75 | fen | 124 | GRA | Present | retained from the source database |
| Turraun Phalaris | Ireland | 53.275 | −7.75 | fen | 62 | GRA | Present | retained from the source database |
| Turraun Typha | Ireland | 53.275 | −7.75 | fen | 62 | GRA | Present | retained from the source database |
| Undrained PSF (Katingan Mentaya Project) | Indonesia | −2.89 | 113.15 | Peat Swamp | 7 | NGRA | Absent-negligible | retained from the source database |
| Undrained peat swamp forest hollow | Indonesia | −2.825 | 111.803 | peat swamp | 18 | NGRA | Absent-negligible | retained from the source database |
| Undrained peat swamp forest hummock | Indonesia | −2.825 | 111.803 | peat swamp | 18 | NGRA | Absent-negligible | retained from the source database |
| Undrained tall sedge mire (UM) | Sweden | 57.13 | 14.75 | bog | 27 | GRA | Present | retained from the source database |
| Vasyuganie field station, Tomsk_RU-VAS-Ridge-hollow complex (Hollow) site | Russia | 56.979 | 82.613 | Bog | 6 | NGRA | Present | re-read from the source publication |
| Vasyuganie field station, Tomsk_RU-VAS-Ridge-hollow complex (Ridge) site | Russia | 56.979 | 82.614 | Bog | 6 | NGRA | Present | re-read from the source publication |
| Vasyuganie field station, Tomsk_RU-VAS-open bog site | Russia | 56.971 | 82.618 | Bog | 9 | Mixed | Present | re-read from the source publication |
| Vasyuganie field station, Tomsk_RU-VAS-ryam site | Russia | 56.976 | 82.609 | Bog | 9 | NGRA | Present | re-read from the source publication |
| Virgin Cottongrass pine bog | Finland | 61.7833 | 24.3167 | bog | 20 | NGRA | Present | re-read from the source PDF |
| Virgin Tall sedge fen (T1) | Finland | 61.7833 | 24.3167 | fen | 21 | GRA | Present | retained from the source database |
| Virgin herb-rich wet flark fen (Puohtiinsuo) | Finland | 62.667 | 30.8333 | fen | 22 | GRA | Present | retained from the source database |
| Virginia wetland site5 | USA | 35.9061 | −76.1569 | Peat swamp | 11 | NGRA | Absent-negligible | retained from the source database |
| Virginia wetland site6 | USA | 35.9061 | −76.1569 | Peat swamp | 11 | NGRA | Absent-negligible | retained from the source database |
| West Kalimantan | Indonesia | 0.53 | 109.5 | peat swamp | 408 | NGRA | Absent-negligible | retained from the source database |
| Xiao Xing’an Mountains – Carex schmidtii seasonal flooded sedge marsh | China | 48.1333 | 128.625 | Marsh | 15 | GRA | Present | retained from the source database |
| Xiaoxing’an Mountains Alnus swamp MCY | China | 48.167 | 128.667 | peat swamp | 15 | NGRA | Present | re-read from the source PDF |
| Xiaoxing’an Mountains birch swamp BH | China | 48.167 | 128.667 | peat swamp | 15 | NGRA | Present | re-read from the source PDF |
| Xiaoxing’an Mountains sedge marsh TC | China | 48.167 | 128.667 | Marsh | 15 | GRA | Present | retained from the source database |
| Xiaoxing’an Mountains – Carex schmidtii freshwater graminoid marsh | China | 48.167 | 128.625 | Marsh | 15 | GRA | Present | retained from the source database |
| Xiaoxing’an Mountains – Mild-thawing permafrost peatland | China | 48.167 | 128.625 | bog | 68 | NGRA | Present | re-read from the source PDF |
| Xiaoxing’an Mountains – No-thawing permafrost peatland | China | 48.167 | 128.625 | bog | 68 | NGRA | Present | re-read from the source PDF |
| Xiaoxing’an Mountains – Severe-thawing permafrost peatland | China | 48.167 | 128.625 | fen | 68 | GRA | Present | retained from the source database |
| Yellowknife collapse wetland | Canada | 62.457 | −114.51 | Fen | 76 | NGRA | Absent-negligible | retained from the source database |
| Yellowknife collapse wetland | Canada | 62.457 | −114.51 | Fen | 70 | GRA | Present | retained from the source database |
| Yellowknife collapse wetland | Canada | 62.457 | −114.51 | Fen | 9 | Uncertain | Unknown | re-read from the source publication |
| Yongqing forestry center, Xiaoxing’an Mountains in Northeast China | China | 48.12 | 128.633 | Bog | 17 | NGRA | Absent-negligible | retained from the source database |
| Yongqing forestry center, Xiaoxing’an Mountains in Northeast China | China | 48.12 | 128.633 | Fen | 17 | NGRA | Unknown | re-read from the source PDF |
| Young Alder-Reed Successional Stand (YA), Estonia | Estonia | 58.216 | 26.7333 | riparian wetland | 18 | GRA | Present | retained from the source database |
| Zackenberg Valley | Greenland | 74.5 | −21 | Minerotrophic tundra fen | 21 | GRA | Present | retained from the source database |
| Zackenberg Valley | Greenland | 74.5 | −21 | Minerotrophic tundra fen | 3 | NGRA | Absent-negligible | retained from the source database |
| Zoige National Wetland Reserve | China | 33.93 | 102.866 | bog | 4 | NGRA | Absent-negligible | retained from the source database |
| Zoige National Wetland Reserve | China | 33.93 | 102.866 | bog; fen | 2 | GRA | Present | retained from the source database |
| Zoige Plateau Seasonal Flooded Marsh site | China | 33.9167 | 102.867 | Natural alpine seasonal-flooded peat marsh | 21 | GRA | Present | retained from the source database |
| Zoige alpine wetlands | China | 33.93 | 102.866 | bog | 3 | GRA | Present | retained from the source database |
| Zoige white dragon river Transition area peatland | China | 34.035 | 102.72 | Fen | 16 | Uncertain | Unknown | re-read from the source publication |
| Zoige white dragon river peatland bulge area peatland | China | 34.035 | 102.72 | Fen | 16 | Uncertain | Unknown | re-read from the source publication |
| Zoige white dragon river peatland hollow area peatland | China | 34.035 | 102.72 | Fen | 16 | Uncertain | Unknown | re-read from the source publication |
| Zoige – Carex muliensis site | China | 33.93 | 102.866 | bog | 1 | GRA | Present | retained from the source database |
| Zoige – Dry hummock | China | 33.93 | 102.866 | bog | 1 | GRA | Present | retained from the source database |
| Zoige – Eleocharis valleculosa site | China | 33.93 | 102.866 | bog | 1 | GRA | Present | retained from the source database |
| Zoigê Plateau – Permanently flooded hollow (P-hollow) | China | 33.92 | 102.82 | Marsh | 12 | NGRA | Present | re-read from the source PDF |
| Zoigê Plateau – Permanently flooded hummock (P-hummock) | China | 33.92 | 102.82 | Marsh | 12 | GRA | Present | retained from the source database |
| Zoigê Plateau – Seasonally flooded hollow (S-hollow) | China | 33.92 | 102.82 | Marsh | 12 | GRA | Present | retained from the source database |
| Zoigê Plateau – Transitional lawn (between P & S wetlands) | China | 33.92 | 102.82 | Marsh | 12 | GRA | Present | retained from the source database |
| e Zoige Wetland Natural Reserve Dry meadow | China | 33.87 | 102.6 | Dry meadow / Degraded peatland | 9 | GRA | Present | retained from the source database |
| e Zoige Wetland Natural Reserve Intact peatland | China | 33.92 | 102.87 | Peatland / Marsh | 10 | GRA | Present | retained from the source database |
| e Zoige Wetland Natural Reserve Wet meadow | China | 33.9 | 102.69 | Wet meadow | 10 | GRA | Present | retained from the source database |
| mixed Sphagnum–Rhynchospora–graminoid community | Canada | 49.1298 | −122.985 | bog | 12 | Mixed | Present | re-read from the source PDF |
| peatlands of Ilomantsi | Finland | 62.767 | 29.833 | bog | 9 | Uncertain | Unknown | re-read from the source publication |
| peatlands of Ilomantsi | Finland | 62.767 | 29.833 | bog | 6 | NGRA | Absent-negligible | retained from the source database |
| peatlands of Ilomantsi | Finland | 62.767 | 29.833 | fen | 3 | GRA | Present | retained from the source database |
| rewetted riparian wetland, Funen | Denmark | 55.22 | 10.29 | Marsh | 21 | GRA | Present | retained from the source database |
